# The vaccine-site microenvironment: impacts of antigen, adjuvant, and same-site vaccination on antigen presentation and immune signaling

**DOI:** 10.1136/jitc-2021-003533

**Published:** 2022-03-11

**Authors:** Max O Meneveau, Pankaj Kumar, Kevin T Lynch, Sapna P Patel, Craig L Slingluff

**Affiliations:** 1 Department of Surgery, University of Virginia, Charlottesville, Virginia, USA; 2 Department of Biochemistry and Molecular Genetics, University of Virginia, Charlottesville, Virginia, USA; 3 Department of Melanoma/Medical Oncology, Division of Cancer Medicine, University of Texas MD Anderson Cancer Center, Houston, Texas, USA; 4 University of Virginia Cancer Center, Charlottesville, Virginia, USA

**Keywords:** melanoma, antigen presentation, vaccination, adjuvants, immunologic, arginase

## Abstract

**Background:**

A goal of cancer vaccines is to induce strong T cell responses to tumor antigens, but the delivery method, schedule, and formulation of cancer vaccines have not yet been optimized. Adjuvants serve to increase the immune response against vaccine antigens. However, little is known about the impact of adjuvants plus antigen and their delivery schedule on the immunologic milieu in the vaccine-site microenvironment (VSME). We hypothesized that antigen processing and presentation may occur directly in the VSME, that adding the toll-like receptor 3 (TLR3) agonist polyICLC (pICLC) would enhance markers of immune activation, and that the immune signatures would be enhanced further by repeated vaccination in the same skin site rather than after multiple vaccines in different skin locations.

**Methods:**

Using RNA sequencing, we evaluated VSME biopsies from patients undergoing subcutaneous/intradermal peptide vaccination against melanoma, with incomplete Freund’s adjuvant (IFA) with or without pICLC. Differential gene expression analyses and gene set enrichment analyses were performed using R. False discovery rate corrected p values <0.05 were considered significant.

**Results:**

We found that addition of peptide antigens to IFA enhanced antigen presentation pathways and a tertiary lymphoid structure gene-signature locally at the VSME. Addition of pICLC to IFA + peptide induced an immunologically favorable VSME 1 week after injection but had little impact on the VSME after three injections, compared with IFA + peptide alone. Repeated same-site injection of IFA + peptide antigens induced a VSME with more dendritic cell activation, Th1 dominance, and TLR adaptor protein gene expression than that induced by injections at different, rotating skin locations.

**Conclusions:**

These data suggest that the vaccine-site itself may be a critically important location contributing to vaccine immunity rather than just the draining lymph node, that IFA induces a favorable VSME with TLR agonist being most beneficial early in the vaccine course, and that same-site injections lead to persistent stimulation of immune pathways that may be beneficial in eliciting antigen specific T cell expansion.

## Background

Immune-mediated control of cancer can be achieved with checkpoint blockade therapy. However, patients without pre-existing T cell responses to their cancer are unlikely to respond.^1^ For these patients, an approach to induce T cell responses is needed. Cancer vaccines hold promise toward this goal; however, their optimal methods of delivery, schedule, and formulation have not yet been defined. For vaccines to be effective in tumor control, a strong antigen-specific T cell response is likely required. One of the most important vaccine components is the adjuvant, which activates dendritic cells (DC) at the vaccine-site and which may serve as an antigen depot. Many vaccine adjuvants are in clinical trials, alone or in combinations, and include, but are not limited to, toll-like receptor (TLR) agonists and lipid emulsions such as incomplete Freund’s adjuvant (IFA). When antigen plus an inflammatory stimulus are co-administered to a cutaneous site, immature DC present the antigen, become activated, and migrate to regional lymph nodes, where they interact with naïve T cells entering the nodes through high endothelial venules (HEV).^2 3^ However, DC activation and maturation can be skewed by the cytokine milieu in which those processes occur^4^; thus, the effects of adjuvants and antigen in the vaccine site may well impact the nature of the DC response, which is critical for subsequent T cell responses. However, very limited human data are available about the impact of adjuvants plus antigen on the immunologic milieu at the vaccine site.

We have shown that repeated injection of IFA at a skin vaccine site (with or without antigen) increased the ratio of Tbet^+^ cells to GATA3^+^ cells in the vaccine-site microenvironment (VSME).^5^ We also found that repeated injection of IFA + peptide antigens induced tertiary lymphoid structures (TLS) containing high endothelial venules (HEV)^6^ and increased the accumulation of antigen-specific T cells at the VSME.^7^ Thus, repeated injection of peptide antigens emulsified in IFA can create a VSME that may support T cell responses to those antigens. However, murine studies have raised concern about the use of IFA, showing that a single injection of a short peptide antigen in IFA induced a chronic inflammatory depot which led to T cell sequestration, dysfunction, and death in the vaccine site.^8^ On the other hand, our subsequent human clinical trials have shown that circulating T cell responses to short peptides were stronger and more durable when IFA was added to peptide antigens + a TLR agonist,^9^ and other work with long peptides also supports more favorable immune responses when IFA was included in the vaccine adjuvant.^10^ Addition of TLR agonists to IFA plus multipeptide vaccines has also enhanced both T cell and antibody responses over IFA alone; but this combination may increase the risk of severe injection site reactions.^11 12^ Also, chronic TLR activation can be immunosuppressive.^13 14^ Together, these findings highlight the need to understand the effects in humans of IFA and TLR agonists on immune-related gene expression locally at the VSME after one or several vaccinations.

In four clinical trials of multipeptide melanoma vaccines, we have evaluated the impact of adjuvants on T cell and antibody responses. The adjuvants have included IFA (Montanide ISA-51, Seppic) alone and IFA plus the TLR3 agonist polyICLC (pICLC, Oncovir) and have incorporated VSME biopsies after one and/or three vaccines. These tissue samples provide a rich source of information, which we believe is the largest human tissue resource available to address questions pertaining to the impact of adjuvants on immunologic responses in the VSME. Previously, we have observed accumulation of activated (CD69^+^) antigen-specific T cells at vaccine sites, as well as formation of TLS in the VSME^7^; thus, we hypothesized that antigen processing and presentation may occur directly in the VSME, rather than only in the draining nodes. Most cancer vaccine trials have incorporated multiple injections over time, usually with subsequent vaccines administered at a different skin location than the prior (rotating vaccines, RV) in order to increase the number of lymph-node basins exposed to antigen.^15 16^ However, we have used repeated same-site vaccination (SSV) in some studies, and RV in others. In recent pilot studies, we found that repeated SSV×3 with peptide plus IFA enhanced expression of DC activation and maturation genes (CD80, CD83, CD86), as well as genes critical to DC licensing (CD40 and CD40-ligand) in the VSME, compared with normal skin.^17^ Interestingly, SSV×3 with peptides in IFA reduced arginase 1 (ARG1), a marker of myeloid suppression. We hypothesized that these effects would be greater after SSV versus RV. Further, we have previously found that adding pICLC to IFA + peptide increased expression of TLR pathway genes (TRIF and MyD88) and Th1 associated genes (IFN-γ, STAT1) in the VSME after one vaccine. Surprisingly, however, we found that SSV×3 with just IFA + peptide induced a much stronger Th1 gene signature^7^ and increased expression of TRIF and MYD88, even without the inclusion of a TLR agonist. We hypothesized that adding pICLC to IFA + peptide for SSV×3 would further enhance TRIF and MYD88, especially after SSV×3, and that other markers of immune activation would be enhanced by adding pICLC to IFA + peptide. Goals of the present study were to address these hypotheses about the impact on the VSME of peptide, SSV versus RV, and addition of pICLC to IFA + peptide, using analyses of gene expression by RNA sequencing (RNA-seq) on 90 vaccine-site biopsies collected from patients enrolled in four clinical trials.

## Methods

### Participants and trials

For the present study, biopsy samples of the VSME were analyzed from participants with stage IIB–IV melanoma, and clinically free of disease, on four peptide vaccine clinical trials: Mel48 (NCT00705640),^7^ Mel58 (NCT01585350),^18^ Mel60 (NCT02126579),^10^ and Mel63 (NCT02425306).^12^ In all of these trials, participants were immunized weekly for 3 weeks (at week (W) 0 (day 1), W1 (day 80), and W2 (day 15)), then every 3 weeks×3. All vaccines included peptide antigens for CD4 T cells, and Mel48, Mel58, and Mel60 included peptide antigens for CD8 T cells. All injections were administered to cutaneous sites (with half of the vaccine injected into subcutaneous tissue and half injected intradermally through a single skin puncture site (sc/id)). Skin biopsies were obtained from normal control skin, and from vaccine sites at W1 and/or W3. In one of these trials (Mel48), an additional sequence of vaccines was administered at one site on a different extremity to induce a VSME solely for the purpose of VSME biopsies, and a subset of those patients were injected only with IFA, and without peptide, at this site. Additional details for each trial are summarized in table 1 and in the supplementary materials.

**Table 1:** Clinical trials and treatment conditions of study samples

Trial	Group	Biopsy week	Peptide antigens	Adjuvant(s)	SSV vs RV	Treatment group	n
Mel48	1A/2A	0	None	None	-*	Normal skin control	3
	1B	1	None	IFA	-	IFA	3
	1C	3	None	IFA	SSV	IFA	4
	2B	1	MELITAC-12.1	IFA	-	IFA + P	5
	2C	3	MELITAC-12.1	IFA	SSV	IFA + P	4
Mel58	2B/2C	1	MELITAC-12.1	IFA + pICLC	-	IFA + pICLC + P	12
Mel60 Part 1	A	1	LPV7 +tet	IFA	-	IFA + P	4
	A	3	LPV7 +tet	IFA	RV	IFA + P	3
	E	1	LPV7 +tet	IFA + pICLC	-	IFA + pICLC + P	6
	E	3	LPV7 +tet	IFA + pICLC	RV	IFA + pICLC + P	6
Mel60 Part 2	E2	1	LPV7	IFA + pICLC	-	IFA + pICLC + P	9
	E2	3	LPV7	IFA + pICLC	SSV	IFA + pICLC + P	14
Mel63	A	1	6MHP	IFA	-	IFA + P	3
	A	3	6MHP	IFA	SSV	IFA + P	3
	C	1	6MHP	IFA + pICLC	-	IFA + pICLC + P	6
	C	3	6MHP	IFA + pICLC	SSV	IFA + pICLC + P	5

*Rows not marked as SSV or RV are from weeks 0–1 so SSV and RV are not applicable.

IFA, incomplete Freund’s adjuvant; LPV7, long peptide melanoma vaccine; MELITAC-12.1, 12 melanoma peptide vaccine; 6MHP, 6 melanoma helper peptide vaccine; P, peptide; pICLC, polyICLC; RV, rotating site vaccination; SSV, same-site vaccination.

Samples from these four trials were grouped by treatment as follows: Control (normal skin; n=3), W1 IFA alone (n=3), W1 IFA + peptide (n=12), W1 IFA + pICLC + peptide (n=33), W3 SSV IFA alone (n=4), W3 SSV IFA + peptide (n=7), W3 SSV IFA + pICLC + peptide (n=19), W3 rotating-site vaccination IFA + peptide (n=3), and W3 rotating-site vaccination IFA + pICLC + peptide (n=6) (table 1). Some of these samples have been included in our prior study,^19^ but the number of samples has been expanded to address the new questions posed in the introduction, which requires comparison to data from some of those earlier samples.

### Tissue collection, RNA processing, and library preparation

Tissue was collected either by 4 mm punch biopsy or by surgical excision of full-thickness skin under local anesthesia. Samples were stored immediately in RNAlater at the bedside. Details of the RNA processing and library preparation methods are described in the online supplemental materials.

10.1136/jitc-2021-003533.supp1 Supplementary data

### RNA-seq

The RNA-seq method used in the present analysis has been previously published^19^ but briefly, the Illumina NextSeq 75 bp High Output sequencing kit with Illumina NextSeq 500 (Illumina, San Diego, California; 75 cycle, single read sequencing) were used according to the manufacturer-recommended procedure. Samples were run on the Illumina NextSeq 500 for single-end sequencing and transferred to the Illumina Base Space interface. Quality control (QC) was performed by assessing the numbers of reads in millions passing filter and the per cent of indexed reads. All runs passed the Illumina QC procedures. The clean reads were mapped with the star aligner to the GENCODE V.27 of the transcriptome and hg38 human genome build. The HTseq^20^ software was used to count aligned reads mapping to each gene.

### Statistical analysis

Differential gene expression analysis was performed using DESeq2 in R (further details provided in online supplemental materials).^21^ Samples were grouped across trials according to the different treatment conditions. Samples were collected from vaccine sites at week 1 or week 3. In some of the trials, samples were collected at both time points for each patient; whereas in other trials, the samples were collected only at one time point. Thus, paired analyses of samples from weeks 1 and 3 would have limited power and were not part of this study. False discovery rate (FDR)-corrected p values (FDR-p) <0.05 were considered significant. Gene set enrichment analysis (GSEA) was performed using the Kyoto Encyclopedia of Genes and Genomes^22^ gene sets to identify enriched biological pathways using the *clusterProfiler* package,^23^ and a custom TLS gene set was created comprised of previously described TLS-associated genes.^17^ The normalized enrichment score (NES) and FDR-adjusted p-value (FDR) was calculated for exploratory gene sets and significance was considered at FDR-p <0.05. For the custom gene set (TLS), the raw p-value was used due to the lack of multiple hypotheses, and p<0.05 was considered significant. Examples of the count matrix and code for the above analyses are provided in the methods section of the supplemental materials document.

## Results

**Figure 1:**
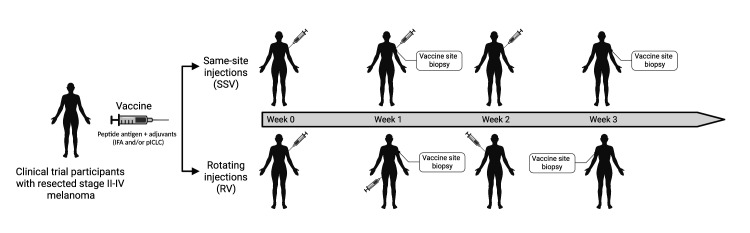
Vaccine injection schema of participants on the Mel48, Mel58, Mel60, and Mel63 clinical trials. Participants with resected stage II–IV melanoma were immunized with a combination of IFA alone, IFA + peptide, or IFA + pICLC + peptide. The peptides included one of MELITAC12.1, 6MHP, or LPV7. Participants underwent either rotating or same-site injection of the vaccine at three time points (weeks 0,1, 2). Tissue from the vaccine site was obtained by either punch biopsy or surgical excision 1 week after the first injection (week 1) and/or 1 week after the third injection (week 3). IFA, incomplete Freund’s adjuvant; pICLC, polyICLC; RV, rotating vaccines; SSV, same-site vaccination.

We performed RNA-seq on a total of 90 samples from the vaccine sites of participants on four melanoma-peptide based vaccine clinical trials. Participants underwent repeated SSV or RV with IFA alone, IFA + peptide, or IFA + pICLC + peptide (figure 1). Biopsies were performed 1 week after the first injection (W1) and 1 week after the third (W3). For participants receiving RV injections, the W3 biopsy was performed at the location of the third injection (W2). We previously reported effects of SSV×3 with IFA + peptide in the VSME but have not examined how these compare to effects of RV×3 with IFA + peptide nor have we reported how the addition of TLR agonist (pICLC) impacts the local immunologic response in the VSME at W3, after repeated injections.^19^

### Peptide antigen is an important driver of the early immune response in the VSME and induces a TLS gene signature

To address our first hypothesis, that antigen processing and presentation may occur directly in the VSME rather than only in the draining nodes, we focused on samples collected 1 week after a single vaccine. This was done because we have rarely observed circulating T cell responses to vaccines at this time point^12 24^ and when we harvested vaccine-draining nodes at W1, we did not detect T cell responses to peptides ex vivo.^18^ Thus, at this early time point, changes in the VSME are likely due to effects of the vaccine in the VSME rather than those induced by circulation of antigen-specific T cells from the vaccine-draining node back to the VSME.

**Figure 2:**
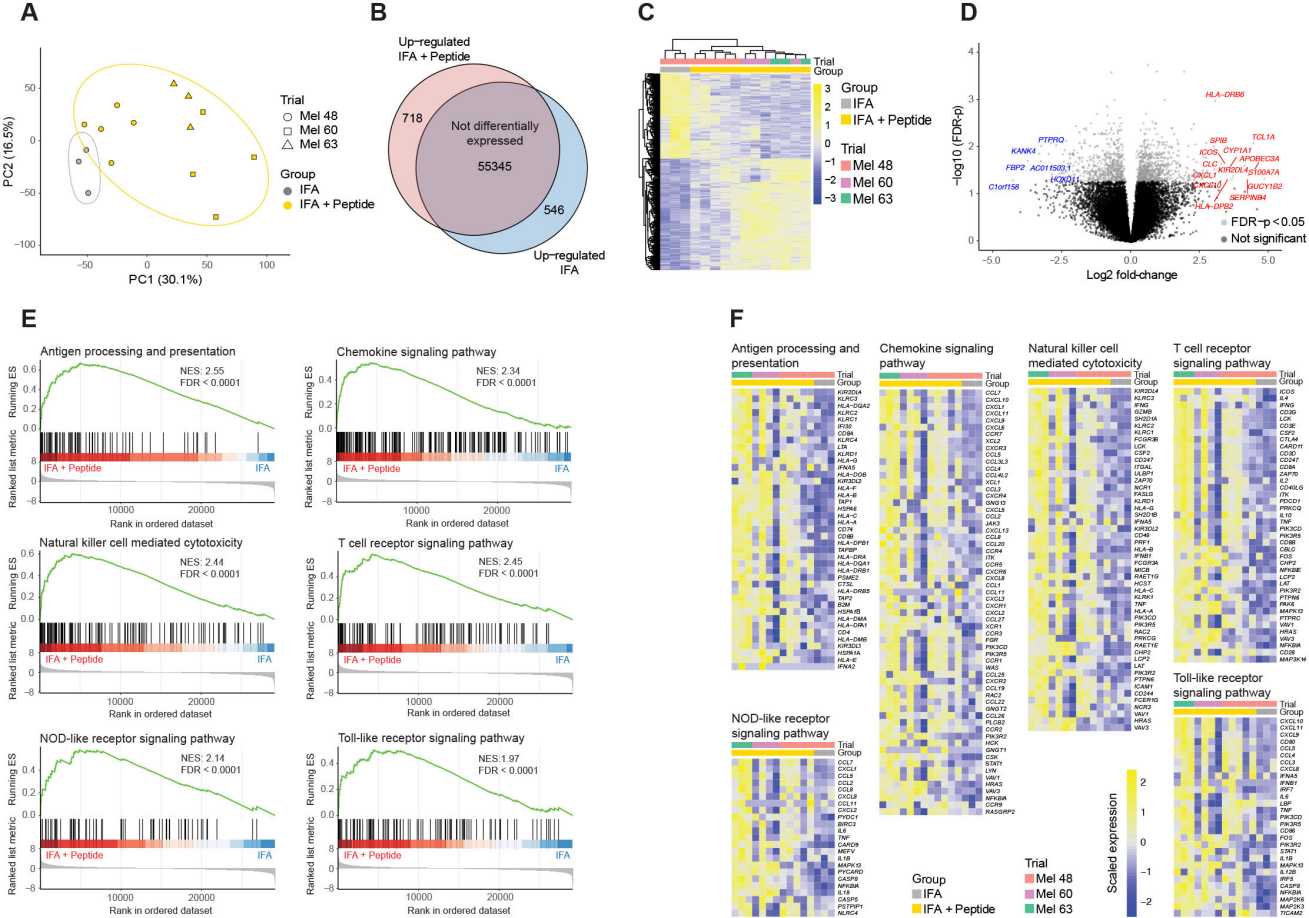
Peptide is a critical component of the early immune response and stimulates antigen presentation in the vaccine-site microenvironment. (A) Principal component analysis demonstrating grouping of the IFA alone and IFA + peptide treated samples. Principal component analysis of PC3 and PC4 are shown in online supplemental figure 1. (B) Results of the differential gene expression analysis showing the number of genes upregulated in the IFA + peptide and IFA alone groups. (C) Heatmap of the differentially expressed genes (DEG’s) with unsupervised clustering of samples. (D) Volcano plot of the DEG’s in gray. Genes labeled in red and blue are in the group of top 20 most differentially expressed by FDR-p <0.05 and ranked by log2 fold change. (E) Selected gene set enrichment analysis plots of highly altered immune related pathways. (F) Heatmaps of the leading-edge genes contributing to the enrichment score in the selected pathways. FDR, false discovery rate; FDR-p, FDR corrected p value; IFA, incomplete Freund’s adjuvant; NES, normalized enrichment score; P, peptide.

To investigate the effect of peptide itself in modulating the early immune response in the VSME, we compared the gene expression profiles of VSME biopsies from W1 of participants immunized with either IFA alone (n=3) or IFA + peptide (n=12). Principal component analysis of the expression profiles revealed grouping by clinical trial and by the treatment condition (figure 2A, online supplemental figure 1). Differential gene expression analysis comparing the IFA alone and IFA + peptide groups revealed 1264 differentially-expressed genes, with 718 upregulated and 546 downregulated in the IFA + peptide group (figure 2B, C). Stratification by clinical trial revealed that participants on the Mel48 trial immunized with IFA + peptide had fewer differentially expressed genes in comparison with IFA alone than those on both Mel60 (peptides=LPV7) and Mel63 (peptides=6 MHP) treated with IFA + peptide compared with IFA alone (online supplemental figures 2 and 3).

**Figure 3:**
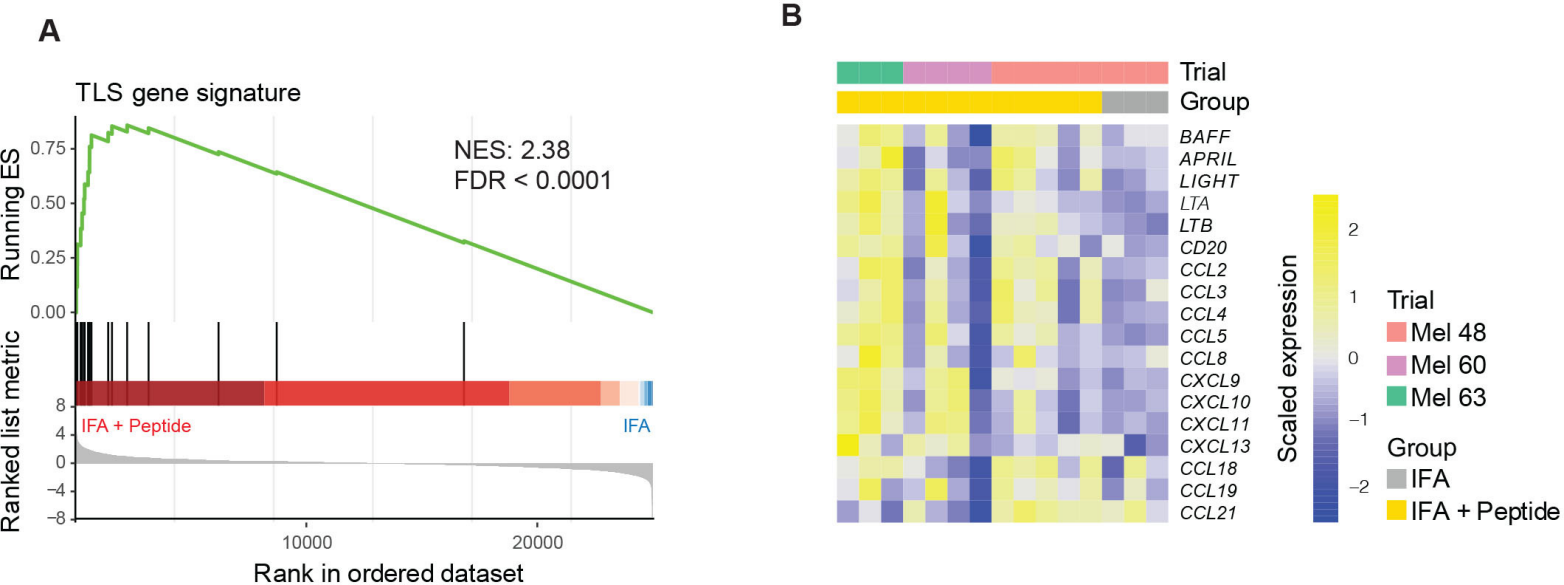
Enrichment of a tertiary lymphoid structure (TLS) gene signature in the vaccine site of participants immunized with IFA + peptide compared with IFA alone. (A) Gene set enrichment analysis plot of the TLS gene signature showing significant enrichment after treatment with IFA + peptide at week 1. (B) Heatmap of the TLS associated gene signature demonstrating low expression of TLS associated genes among the IFA alone treated participants. FDR, false discovery rate; FDR corrected p value; IFA, incomplete Freund’s adjuvant; NES, normalized enrichment score; P, peptide.

We then examined the top 20 most significantly altered genes between those receiving IFA alone and IFA + peptide (as measured by the log2 fold change and FDR-p <0.05). This revealed that the addition of peptide to IFA induced higher expression of genes associated with DC maturation (APOBEC3A, SERPINB4), NF-kB regulation (TCL1A), antigen processing and presentation (S100A7A), chemokines (CXCL10, CXCL1), and T cell induction and homing (CXCL10, ICOS1) (figure 2D, online supplemental table 1). Furthermore, numerous HLA genes were upregulated in the IFA + peptide group (HLA-DRB6, HLA-DQA2, HLA-B, HLA-H, HLA-C, HLA-F, HLA-DPB1, HLA-DQA1, and HLA-DPB2. Log2 FC>1 FDR-p <0.05), suggesting that antigen presentation may be enhanced in the vaccine site at this early time point.

We then performed GSEA to identify the pathways affected by peptide in combination with IFA. Using KEGG gene sets, 44 enriched pathways were revealed, many of which were immune related (online supplemental table 2). Antigen processing and presentation was highly enriched in the IFA + peptide group (NES: 2.55, FDR-p <0.0001), further supporting the above finding that the VSME is an important site of antigen presentation early after vaccination and suggesting that T cell responses may be generated locally in the VSME (figure 2E, F). This is further supported by the finding that chemokine signaling, natural killer cell mediated cytotoxicity, and T cell receptor signaling were each also significantly enriched (FDR-p <0.0001) in the VSME 1 week after immunization with IFA + peptide.

We have previously reported that TLS have been observed in the vaccine sites of participants immunized with peptide and IFA.^6^ These structures are comprised of organized clusters of B and T cells, as well as mature DC and high endothelial vasculature expressing peripheral node addressin and are thought to drive T cell recruitment and expansion of antigen-specific T cell responses.^25–29^ Having found evidence that combinations of peptide and IFA stimulate antigen presentation locally at the VSME, we hypothesized that the peptides are critical in driving TLS formation. To address this question, we performed GSEA using a previously described^17^ TLS-associated gene set (BAFF, APRIL, LIGHT, LTA, LTB, CD20, CCL2, CCL3, CCL4, CCL5, CCL8, CXCL9, CXCL10, CXCL11, CXCL13, CCL18, CCL19, CCL21). This revealed significant enrichment of the TLS associated gene signature in the IFA + peptide group compared with the IFA alone group (figure 3A, B).

### Impact of SSV and addition of pICLC on DC activation, maturation, and licensing

**Figure 4:**
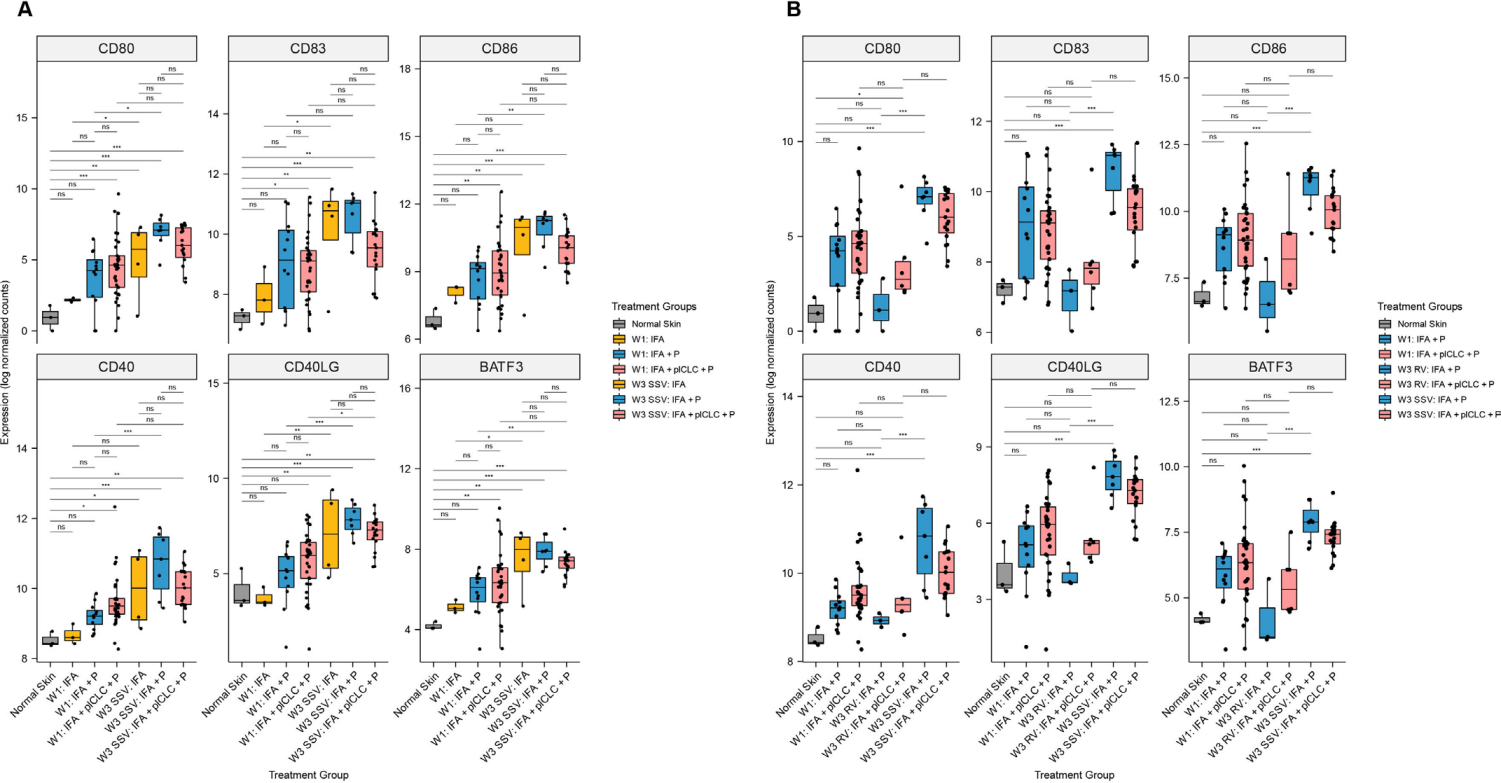
Expression of dendritic cell maturation, activation, and licensing genes in the vaccine-site microenvironment according to treatment group and injection strategy. (A) Comparisons demonstrating that pICLC increases the expression of dendritic cell maturation, activation, and licensing genes early after injection but does not significantly increase their expression compared with week 1 after repeated same-site injection. (B) Comparisons demonstrating that expression of dendritic cell maturation, activation, and licensing genes is lower after rotating vaccine injections compared with same-site injections. Boxplots denote the median and IQR, and boxes are colored according to vaccine formulation. Expression is shown as log normalized counts. Total n=90 samples. All p-values were FDR-corrected. *FDR-p <0.05, **FDR-p <0.01, ***FDR-p <0.001, ****FDR-p <0.0001. FDR, false discovery rate; IFA, incomplete Freund’s adjuvant; ns, not significant; P, peptide; pICLC, polyICLC; RV, rotating-site vaccination; SSV, same-site vaccination; W1, week 1; W3, week 3.

To address our hypothesis that the TLR3 agonist pICLC would enhance markers of immune activation in the VSME, we compared gene expression in the VSME induced after one or three vaccines to normal skin. At W1, 12 and 33 patients were evaluable after vaccination with IFA + peptide and IFA + pICLC + peptide, respectively. Compared with normal skin, vaccination with IFA + pICLC + peptide enhanced expression of DC activation (CD80, CD86), maturation (CD83), and licensing (CD40) genes as well as BATF3 (a marker of DC cross-presentation) at W1, while IFA + peptide alone did not (figure 4A). However, expression levels of those genes, and of CD40L, were similar for IFA + peptide and IFA + pICLC + peptide. Interestingly, SSV×3 with IFA + pICLC + peptide did not enhance expression of CD80, CD86 or CD83 (figure 4A) compared with SSV×3 with IFA + peptide alone. FDR-corrected p values for each comparison in figure 4A are shown in online supplemental table 2.

At W3, VSME biopsies were evaluable for 26 patients after SSV×3. For those vaccinated with IFA + peptide only, SSV×3 enhanced expression of five of six DC-associated genes compared with W1 (CD80, CD86, CD40, CD40L, BATF3). However, among those vaccinated with IFA + pICLC + peptide, SSV×3 significantly enhanced expression only of CD40L (figure 4A). Also, in contrast to our hypothesis that adding pICLC to IFA + peptide would enhance the favorable immune signaling of IFA + peptide after SSV×3, expression of all six of these genes trended lower after SSV×3 with addition of pICLC (figure 4A).

At W3, VSME biopsies were evaluable for nine patients after RV×3. To investigate the effects of RV versus SSV, we first compared the expression of these DC-associated genes for patients who received IFA + peptide after RV×3 versus SSV×3: SSV×3 significantly enhanced expression of all six genes compared with RV×3, whereas the expression after RV×3 was not different than normal skin (figure 4B). Similarly, for those vaccinated with IFA + pICLC + peptide, SSV×3 was compared with RV×3: the trends were in the same direction as with IFA + peptide for all six genes, but the differences were not significant (figure 4B). FDR-corrected p-values for each comparison in figure 4B are shown in online supplemental table 3.

### Th1 gene expression is selectively enhanced by SSV with IFA + peptide, but not by addition of TLR agonist

To address our third hypothesis—that immune signatures in the VSME would be enhanced further by repeated vaccination in the same skin site rather than after multiple vaccines in different skin locations—we compared expression of key immune related genes in the VSME after SSV×3 compared with just one vaccine and also compared with RV×3. We focused on a subset of Th1-associated genes in the ‘immunologic constant of rejection’,^30^ especially the transcription factor driving Th1 differentiation (TBX21/tbet), genes representing CD4^+^ and CD8^+^ T cells (CD8A, CD4), and the genes for interferon-γ (IFN-γ) and STAT1. In prior work, we found, by immunohistochemistry of the VSME induced by IFA + peptides, that the ratio of Tbet^+^ (Th1) to GATA3^+^ (Th2) cells in the VSME increased after SSV×3 compared with normal skin and compared with the VSME after one vaccine.^5^ Also, prior evaluation of a small number of VSME samples by RNA-seq showed that expression of TBX21, STAT1, and IFN-γ were all enhanced significantly in the VSME after SSV×3, compared with normal skin and compared with one vaccine.^19^ In contrast, expression of transcription factors GATA3 and RORC (Th17) were decreased after SSV×3, suggesting a shift to a Th1-dominant VSME. In the present study, we asked whether these findings would be validated in a larger data set, and whether these changes induced after SSV×3 are also induced by RV×3, either for IFA + peptide, or when the TLR3 agonist pICLC is added to IFA + peptide.

**Figure 5:**
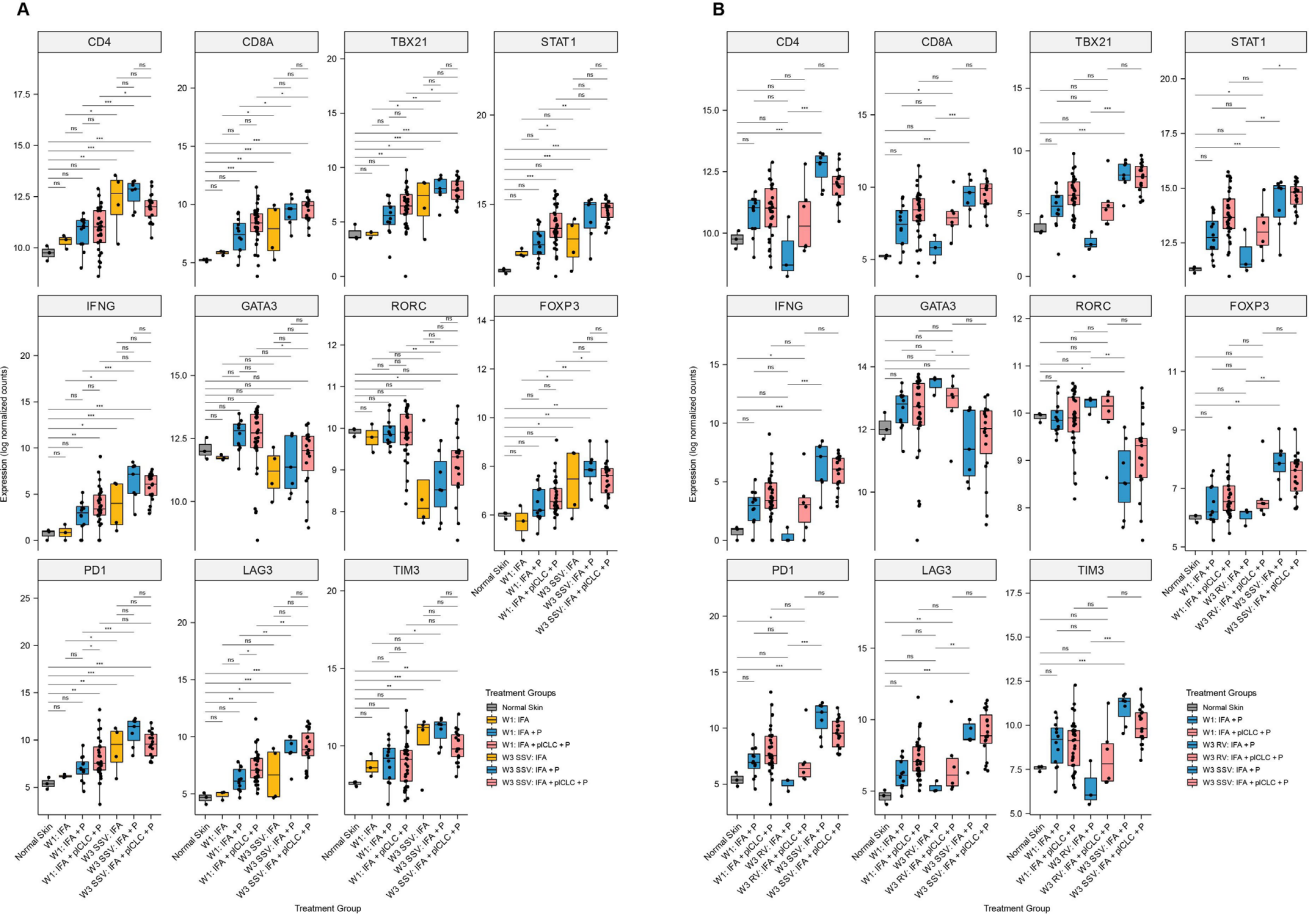
Expression of selected T cell transcription factor and exhaustion marker genes in the vaccine-site microenvironment according to treatment group and injection strategy. (A) Comparisons demonstrating the effect of pICLC at week 3 compared with week 1 after repeated same-site injection. (B) Comparisons after rotating vaccine injections compared with same-site injections. Boxplots denote the median and IQR, and boxes are colored according to vaccine formulation. Expression is shown as log normalized counts. Total n=90 samples. All p values were FDR-corrected. *FDR-p <0.05, **FDR-p <0.01, ***FDR-p <0.001, ****FDR-p <0.0001. FDR, false discovery rate; IFA, incomplete Freund’s adjuvant; IFN, interferon; ns, not significant; P, peptide; pICLC, polyICLC; RV, rotating-site vaccination; SSV, same-site vaccination; W1, week 1; W3, Week 3.w

SSV×3 increased expression of CD4 and CD8A at W3 compared with W1 for participants immunized with IFA + peptide and for those receiving IFA + pICLC + peptide (figure 5A). However, neither CD4 nor CD8A were increased at W3 by addition of pICLC to IFA + peptide after SSV×3 (figure 5A). The Th1 transcription factor TB×21 (Tbet) and the Th1-associated genes STAT1 and IFN- γ were all more highly expressed at W3 after SSV×3 with IFA + peptide than at W1, confirming the findings from our smaller initial data set.^19^ There were trends in the same direction for TBX21, STAT1, and IFN- γ for SSV×3 with IFA + pICLC + peptide; however, only TB×21 increased significantly (figure 5A). Expression of GATA3 was equivalent among all treatment groups except in IFA + pICLC + peptide at W3 (SSV×3), where it was significantly lower than at W1. On the other hand, expression of RORC and the regulatory T cell transcription factor, FOXP3, were similar after SSV×3, with either IFA + peptide or IFA + pICLC + peptide, though RORC was lower than at W1, and FOXP3 higher than at W1, for SSV×3 with either adjuvant combination (figure 5A).

To assess the impact of the site of repeated vaccines on expression of these genes, we compared expression in the VSME induced after vaccination with IFA + peptide, or IFA + pICLC + peptide, after RV×3 versus SSV×3. For patients vaccinated with IFA + peptide, expression of CD4, CD8A, TBX21, STAT1, IFN- γ, and FOXP3 were all significantly increased with SSV compared with RV. In contrast, expression of GATA3 and RORC were significantly lower with SSV versus RV (figure 5B). Similar weak trends were observed for those vaccinated with IFA + pICLC + peptide, but only STAT1 was significantly increased with SSV×3 compared with RV×3. IFA + peptide at RV×3 induced no significant changes compared with normal skin for these eight genes, while IFA + pICLC + peptide with RV×3 induced significant increases in CD8A, STAT1 and IFN- γ compared with normal skin, but no significant differences for the other five genes in this group (figure 5B). FDR-corrected p values for each comparison in figure 5A and B are shown in online supplemental tables 2,3, respectively.

Having addressed the three principal hypotheses for this study, we also explored the impact of vaccine strategies on induction of checkpoint genes, TLR adaptor protein expression, and expression of ARG1, each of which can drive or regulate T cell-mediated immunity.

### Checkpoint genes are selectively enhanced by SSV with IFA + peptide, but not when TLR agonist is added

Activated T cells upregulate expression of PD1, LAG-3 and TIM3,^31^ but these genes also may be markers of T cell exhaustion. In our prior work with a small number of samples, we found that IFA + peptide at SSV×3 increased expression of PD-1, LAG3, and TIM3 compared with normal skin.^19^ Here we assessed the impact of pICLC on these genes and the impact of SSV versus RV. We found that PD1, LAG3, and TIM3 were all more highly expressed at W3 (after SSV×3) in the IFA + peptide group compared with W1, but that only LAG3 was increased in the IFA + pICLC + peptide group after SSV×3 (figure 5A). For IFA + peptide groups, PD1, LAG3, and TIM3 expression after RV×3 were not different from normal skin and were all significantly lower than after SSV×3 (figure 5B). However, for the IFA + pICLC + peptide groups, expression of PD1 and LAG3 in the VSME were higher after RV×3 than in normal skin, but comparing SSV×3 to RV×3, there were only non-significant trends toward higher expression of these checkpoint genes (figure 5B).

### The impact of adding TLR agonist to IFA and peptide on expression of TLR adaptor protein genes

**Figure 6:**
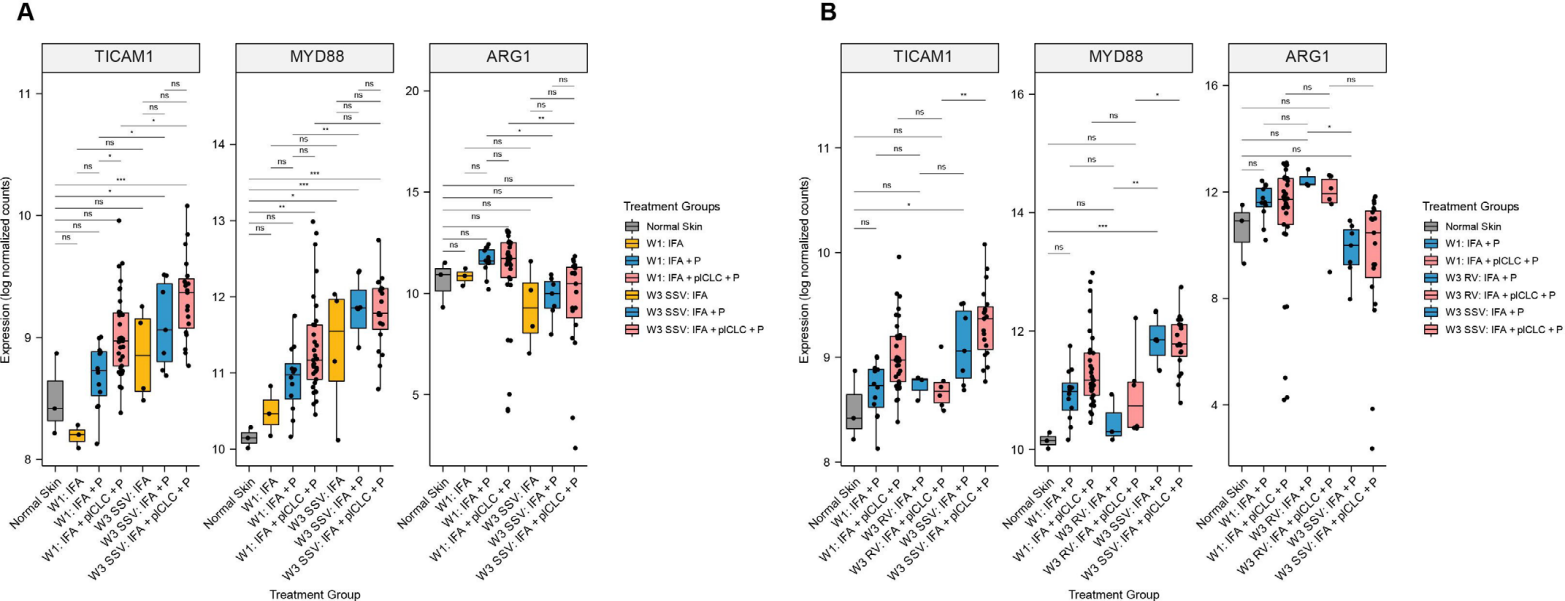
Expression of TLR pathway adaptor protein genes and arginase-1 (ARG1) in the vaccine-site microenvironment according to treatment group. (A) Comparisons at week 3 compared with week 1 after repeated same-site injection. (B) Comparisons after rotating vaccine injections to same-site injections. Boxplots denote the median and IQR, and boxes are colored according to vaccine treatment delivered. Expression is shown as log normalized counts. Total n=90 samples. All p values were FDR-corrected. *FDR-p<0.05, **FDR-p <0.01, ***FDR-p <0.001, ****FDR-p <0.0001. FDR, false discovery rate; IFA, incomplete Freund’s adjuvant; ns, not significant; P, peptide; pICLC, polyICLC; RV, rotating-site vaccination; SSV, same-site vaccination. W1, week 1; W3, week 3.

In our prior pilot study, we found that SSV×3 with IFA + peptide significantly increased signaling through the TLR pathway via TICAM1 and MYD88 expression, compared to IFA + peptide at W1, as well as to normal skin.^19^ In the current larger data set, that prior finding was validated (figure 6A). We hypothesized that pICLC, a TLR agonist, would further enhance expression of TICAM1 and MYD88. Surprisingly, TICAM1 and MYD88 differ in the VSME induced after SSV×3 with either IFA + pICLC + peptide or IFA + peptide alone (figure 6A). The expression of TICAM1, but not MYD88, increased significantly with IFA + pICLC + peptide at W3 compared with W1 (figure 6A). After RV×3, neither was different from normal skin, and MYD88 was expressed at significantly lower levels after RV compared with SSV×3 (figure 6B). For the IFA + pICLC + peptide group, both MYD88 and TICAM1 were significantly higher with SSV×3 compared with RV. FDR-corrected p values for each comparison in figure 6A, B are shown in online supplemental tables 2,3, respectively.

### ARG1 expression is decreased after repeated SSV compared with RV but not affected by the addition of pICLC to IFA and peptide

A barrier to effective cancer vaccines is the suppression of T cell responses and function. One driver for this T cell dysfunction is the presence of myeloid derived suppressor cells,^32^ whose suppressive effect is thought to be mediated in large part by ARG1. Our prior pilot study showed that a single injection of IFA and peptide does not alter the expression of ARG1 in the VSME at W1 but that repeated SSV×3 significantly decreased ARG1 expression in the VSME.^19^ We hypothesized that adding pICLC may increase this reduction in ARG1 expression, and that SSV×3 would more effectively depress ARG1 in the VSME compared with RV. We found that ARG1 expression trended toward slightly higher expression after one vaccine with IFA + peptide or IFA + pICLC + peptide, and that after SSV×3, ARG1 expression was significantly lower than at W1 with either vaccine adjuvant (figure 6A). However, ARG1 expression was significantly lower in the IFA + peptide and IFA + pICLC + peptide groups after SSV×3 compared with W1. ARG1 was significantly lower after SSV×3, than after RV×3, after vaccination with IFA + peptide (figure 6B). For those vaccinated with IFA + pICLC + peptide, there was a trend toward lower ARG1 expression after SSV×3, compared with RV×3, but the difference was not significant (FDR-p=0.108).

## Discussion

The aim of cancer vaccines is to produce robust T cell responses that are both durable and antigen-specific, but the optimal formulation of these vaccines has yet to be defined. Adjuvants are a critical component in this formulation, and TLR agonists and IFA have shown promise as effective adjuvants in a number of human clinical trials.^33–36^ In a smaller pilot study of the VSME, we found that repeated SSV with peptide plus IFA decreased ARG1 expression, upregulated markers of DC activation and CD40L, supported a Th1-dominant immune response and induced a gene signature associated with formation of TLS in the VSME.^19^ The present study validates those prior findings in a larger data set. Most importantly, however, we examined the impact of the peptide itself in the early immune signaling events within the VSME. We found that pathways responsible for antigen presentation, T cell recruitment, T cell expansion, and TLS formation were significantly enriched by the presence of peptide, suggesting that antigen presentation may occur in the VSME, independent of events in the vaccine-draining lymph node, and that the VSME is an important site for T cell activation and expansion early after immunization. We also found that the identity of the peptides themselves may generate distinct immune signatures in the VSME. Addition of long peptides (6MHP, LPV7) to IFA appeared to have a greater impact on VSME gene expression than addition of the shorter peptides (12MP), an effect that may relate to induction of helper T cell responses with the longer peptides. Commonly, cancer vaccines are injected at a different skin site with each administration (RV) and many studies do not explicitly report the placement of subsequent vaccines. In the present study, we found that repeated SSV favorably modulated expression of many critical immune pathway genes compared with RV. In prior work, we had found that addition of the TLR agonist pICLC to IFA + peptide favorably modulated expression of some of these key immune pathway genes compared with IFA + peptide^19^; however, our hypothesis that the benefits of pICLC would be observed also after three vaccines was not supported by the present data. For most of these genes, the most dramatic impact was observed with IFA + peptide alone after three same-site vaccines, whereas the impact was similar or slightly less when pICLC was included. These findings support the value of IFA + peptide for conditioning the VSME to support antigen processing and presentation locally in the VSME and suggest that addition of pICLC with the first vaccine may enhance early immune signaling but that repeated use may not be required.

It is generally accepted that peptide antigens are processed by DCs which then migrate to lymph nodes and become professional antigen presenting cells (APCs).^2 3^ There, they interact with immature T cells and stimulate antigen specific cells to clonally expand. Our finding that antigen presentation, T cell receptor signaling, and chemokine signaling were significantly enriched in the VSME by the presence of peptide indicates a possible role for the vaccine site itself as a critical location of T cell activation early after immunization. This is further supported by the finding that a well-characterized TLS gene signature was significantly increased by the presence of peptide at this early time point. TLS are lymph-node like aggregates that form in peripheral tissues near sites of inflammation and help to generate adaptive immune responses.^37^ These structures in the VSME may support antigen presentation and clonal expansion of antigen specific T cells locally. This could therefore contribute significantly to the immune response generated by vaccines and provide further rationale for repeated SSV.

It is not known whether RV or SSV is optimal. Murine work has suggested that a higher number of lymph node basins exposed to antigen is correlated with stronger antigen specific T cell responses. However, that work did not address the effects of sequential, long-term repeated vaccination. Our group and others^38^ have shown that repeated injections in the same site can generate potent and sustained immune responses. Here, we demonstrated that markers of immune activation are generally not differently expressed (but trend higher) compared with normal skin after just one injection of IFA and peptide. However, SSV×3 with this treatment resulted in significant alterations in gene expression and an immunologically favorable VSME, while RV did not. We have previously demonstrated that cutaneous infiltration of immune cells at the vaccine site is transient and that longer-term exposure to antigen by repeated injection can lead to increased Th1 cell recruitment and formation of TLS in the VSME.^5 19^ These prior findings are supported by the data presented herein, and indicate that repeated SSV results in persistent stimulation of immune pathways that may be beneficial in eliciting antigen specific T cell expansion. Future studies are needed to elucidate whether gene signatures in the VSME accurately identify immune responders. Some early findings do suggest that the VSME of patients with systemic immune responses to peptide vaccine may be distinct from non-responders (manuscript in preparation), and additional studies are in progress.

Clinical trials in humans have demonstrated that the combination of pICLC and IFA produces robust antigen specific T cell and antibody responses when used as adjuvants in peptide-based vaccines.^39 40^ It is believed that the two adjuvants are effective together because IFA provides an inflammatory depot which slowly releases peptide and recruits APCs, while TLR agonists stimulate APCs and increase expression of inflammatory cytokines and co-stimulatory molecules leading to tumor-specific cellular and humoral immune responses.^41 42^ However, IFA + peptide induces antigen processing and presentation and enhanced TLR signaling (figure 2E, F), as well as expression of a range of TLS-associated chemokines in the VSME (figure 3) after one vaccine, and can induce TLS formation and reduce ARG1 in the VSME after three vaccines.^6 19^ Thus, the impact of IFA as an adjuvant has been underestimated, as it appears to have effects beyond a simple depot.

Nonetheless, our group and others have found that T cell and antibody responses to IFA-containing vaccines are further enhanced by addition of a TLR agonist.^39 43^ In our prior pilot study of vaccine sites, we found that addition of pICLC to IFA + peptide in the first vaccine enhanced expression TBX21, IFNG, and MYD88 at W1, compared with normal skin, when IFA + peptide alone did not. Thus, we hypothesized expression of key immune signaling pathway genes would be enhanced after SSV×3 by adding pICLC to IFA + peptide. It was surprizing in the present study that genes related to DC function and T cell responses in fact were mostly *not* more favorably enhanced after SSV×3 with IFA + pICLC + peptide compared with the same treatment at W1. It has been reported that sustained TLR stimulation can hinder vaccine immune responses by stimulating immune tolerance and T cell dysfunction, a potential explanation for these findings.^13 14^ The interplay between pro-inflammatory and anti-inflammatory TLR signaling requires further study, as it remains unclear whether repeated SSV with TLR agonist in combination with IFA is an optimal adjuvant strategy. It is possible that when utilizing repeated same-site injections, the first vaccine should include peptide, IFA, and pICLC with subsequent injections formulated with peptide and IFA alone, to avoid some of the potential immunosuppressive effects of chronic TLR stimulation.

## Conclusions

These data significantly expand our knowledge of the VSME created over time by peptide, IFA, and TLR agonists, and the impact of SSV. These data provide new evidence that the vaccine site itself can support antigen presentation locally, suggesting that clonal antigen specific T cell expansion may occur there, likely supported by the creation of TLS, which are most prominent after repeated SSV. Adding pICLC to the first vaccine may favorably modulate expression of key immune signaling genes, but these data do not support further enhancement of the VSME by repeated injection of pICLC at the same site. Future directions will include analyses of correlations between VSME immune signaling and systemic immune responses to vaccines. Also, further study of immune signaling at the VSME as well as the draining nodes may help to reveal improved strategies for vaccine formulation and delivery.

## Data Availability

Data are available upon reasonable request.
