# Supplementary material for: The vaccine-site microenvironment: impacts of antigen, adjuvant, and same-site vaccination on antigen presentation and immune signaling
Source: J Immunother Cancer. 2022 Mar 11;10(3):e003533. doi: 10.1136/jitc-2021-003533 (PMC8919469; doi:10.1136/jitc-2021-003533)
Supplement: Supplementary data [file jitc-2021-003533supp001.pdf]

The vaccine-site microenvironment: impacts of antigen, adjuvant, and same-site vaccination on antigen presentation and immune signaling

SUPPLEMENTARY MATERIALS

Max O. Meneveau[1], Pankaj Kumar[2], Kevin T. Lynch[1], Sapna P. Patel[3], Craig L. Slingluff, Jr[1,4]

[1] Department of Surgery, University of Virginia, Charlottesville, VA, United States
[2]Department of Biochemistry and Molecular Genetics, University of Virginia, Charlottesville, VA, United States
[3] Department of Melanoma/Medical Oncology, The University of Texas M.D. Anderson Cancer Center, Houston, TX, United States
[4] University of Virginia Cancer Center, Charlottesville, VA, United States

SUPPLEMENTARY FIGURES

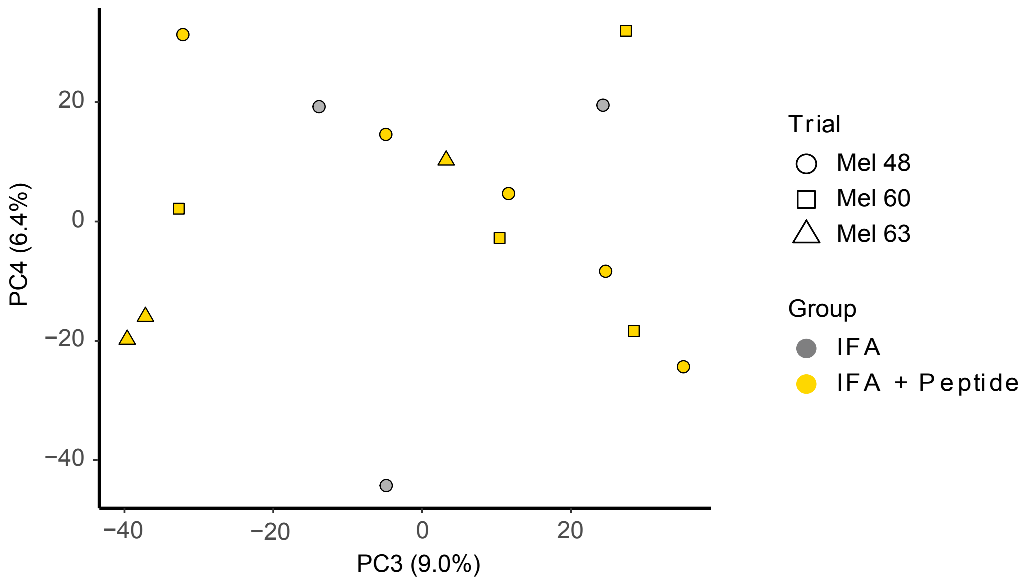

Supplementary Figure 1. Principal component analysis demonstrating PC3 and PC4 of the IFA alone and IFA + peptide treated samples. Samples did not group by trial or treatment in the PC3/PC4 domain.

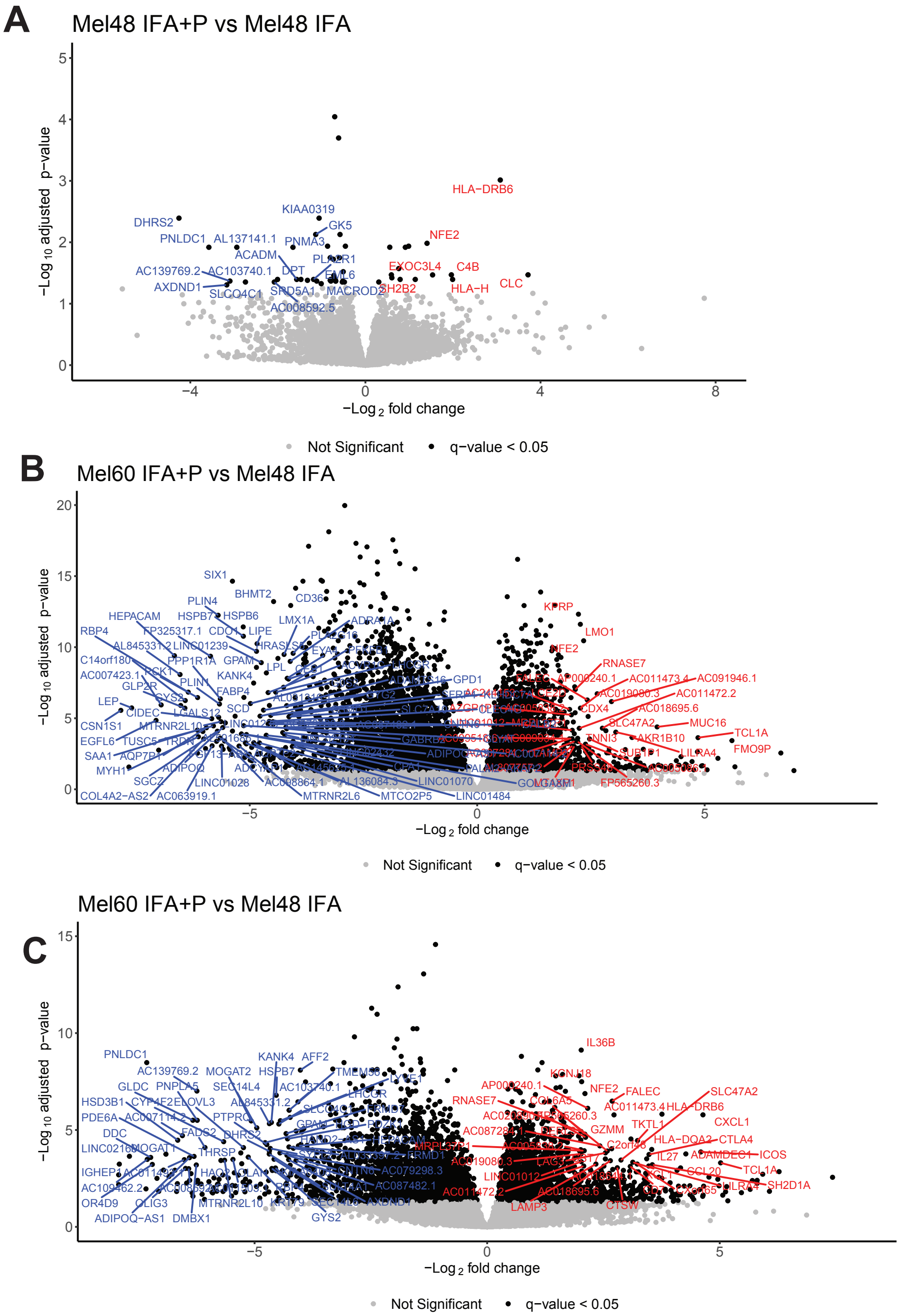

Supplementary Figure 2. Volcano plots of differentially expressed genes in the comparisons between samples from participants treated with IFA + Peptide from each clinical trial vs IFA alone from Mel48.

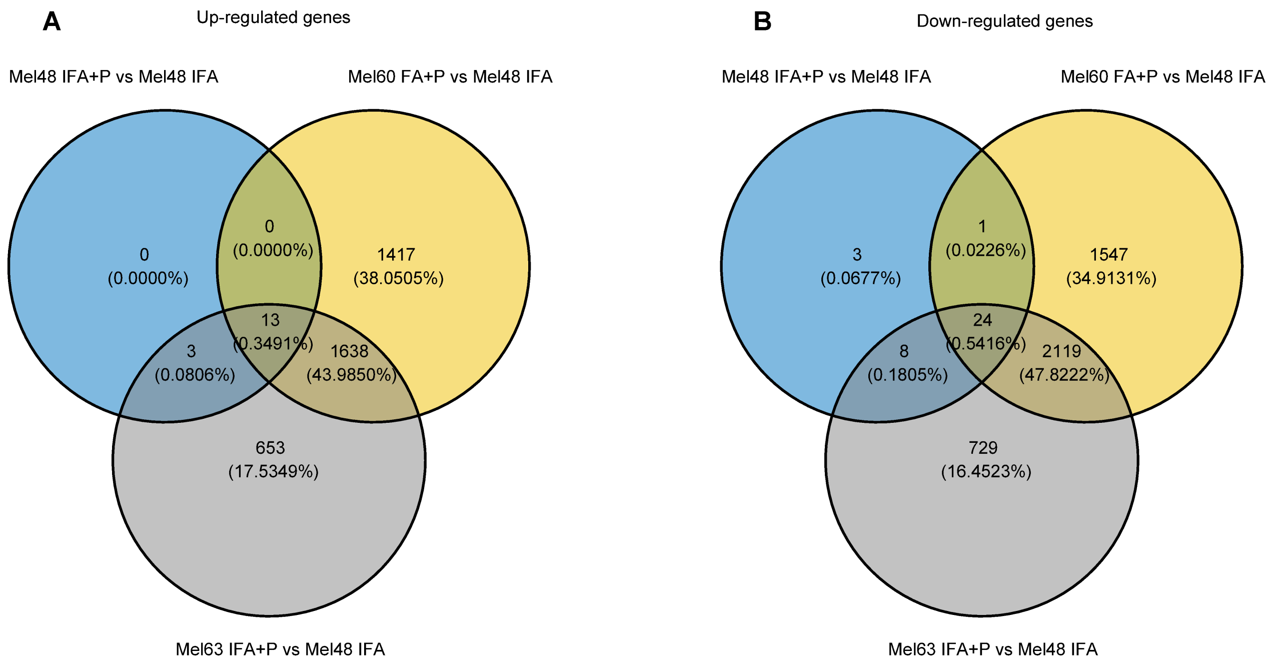

Supplementary Figure 3. These venn diagrams demonstrate the overlap in up **(A)** and down **(B)** regulated genes in the comparison of IFA+P vs IFA alone for each trial. IFA + P vs IFA alone samples from participants on Mel48 had relatively few differentially expressed genes compared the comparisons between Mel48 vs IFA+P from Mel63 and Mel64.

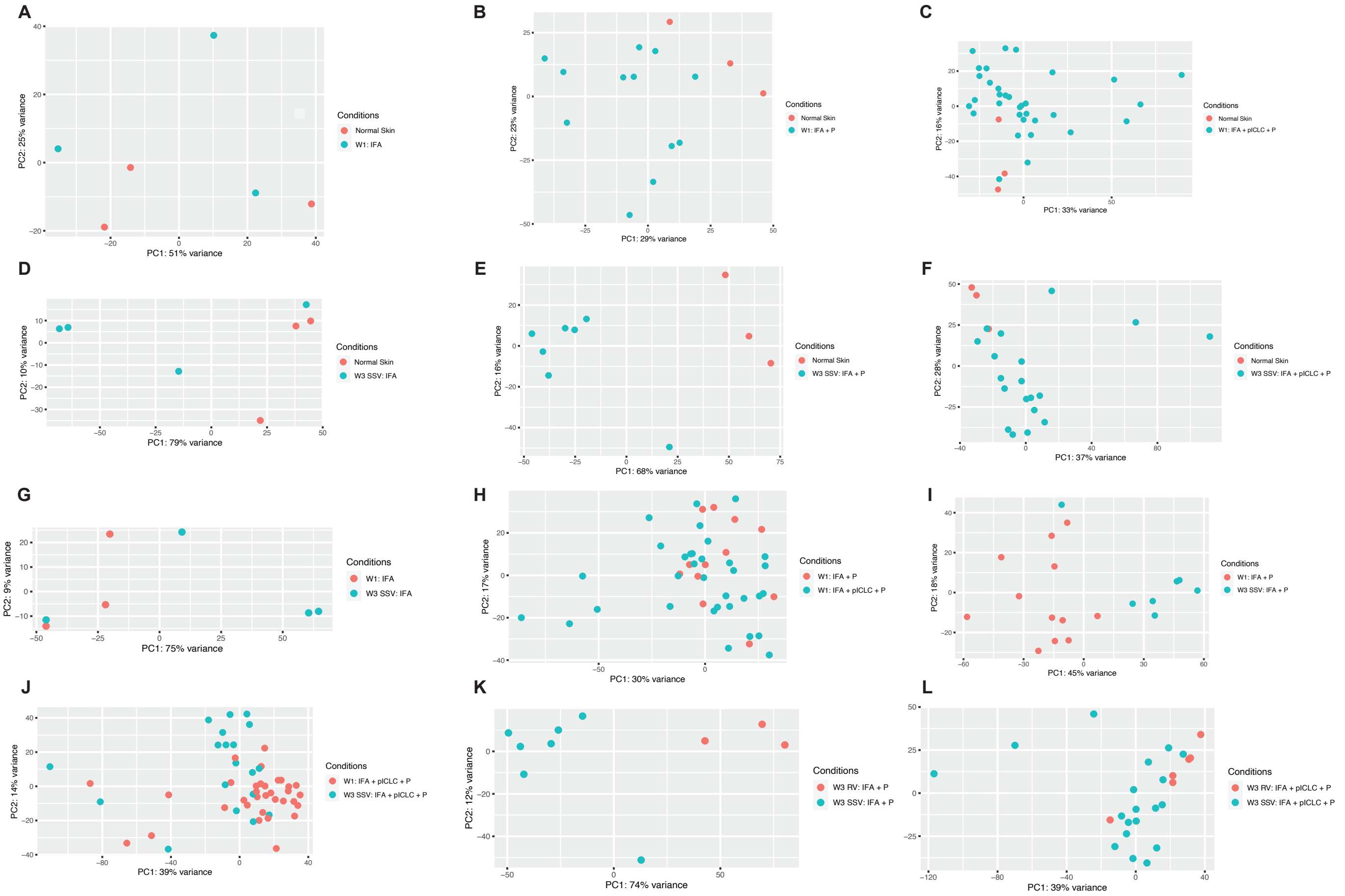

Supplementary figure 4. Principal component analysis (PCA) plots of all contrasts presented in figures 4, 5, and 6 of the main manuscript. W1: IFA vs W1: IFA + P PCA is shown in figure 2 of the main manuscript. IFA = Incomplete Freund's Adjuvant, pICLC = poly-ICLC, P = Peptide, PC = Principal Component.

SUPPLEMENTARY TABLES

Supplementary Table 1. Top 20 most highly differentially expressed genes in IFA + peptide versus IFA alone

Gene Symbol	Gene Name	log2Fold Change	Raw P-value	FDR-p	Function
**GUCY1B2**	guanylate cyclase 1 soluble subunit beta 2 (pseudogene)	4.864	0.002	0.044	Signal transduction mediated by Nitric Oxide [13]
**APOBEC3A**	apolipoprotein B mRNA editing enzyme catalytic subunit 3A	4.504	0.001	0.028	Promotes M1 macrophage differentiation, expressed by mature DC's [14]
**TCL1A**	TCL1 family AKT coactivator A	4.465	0.000	0.008	Transcriptional co-regulator with NF-κB [15]

**S100A7A**	S100 calcium binding protein A7A	4.281	0.002	0.037	Antigen processing and presentation, chemoattractant for CD4-T cells [16]
**SERPINB4**	serpin family B member 4	4.241	0.003	0.046	DC maturation, protection against killing by CTL, Antigen cross-presentation [17]
**KIR2DL4**	killer cell immunoglobulin like receptor, two Ig domains and long cytoplasmic tail 4	3.726	0.002	0.039	Promoter of NK cell immunity, binds peptide-bound HLA-G [18]
**CXCL10**	C-X-C motif chemokine ligand 10	3.680	0.002	0.045	Stimulates chemotaxis, differentiation, and activation of peripheral immune cells, homing receptor ligand for T cells [19]
**CYP1A1**	cytochrome P450 family 1 subfamily A member 1	3.610	0.001	0.025	Mediates chronic immune response by inhibition of the aryl hydrocarbon receptor (AHR) [20]
**SPIB**	Spi-B transcription factor	3.510	0.000	0.009	Promotes differentiation into plasmacytoid DC's [21]
**HLA-DPB2**	major histocompatibility complex, class II, DP beta 2 (pseudogene)	3.508	0.003	0.046	Function in antigen presentation and immune activation [22]
**ICOS**	inducible T cell co-stimulator	3.417	0.000	0.021	Broad function in the adaptive immune response, including induction, maintenance, and homing of T-follicular helper cells, and stimulation of Th1 and Th2 responses [23]
**CLC**	Charcot-Leyden crystal galectin	3.283	0.000	0.021	Maintains function of Treg cells [24]
**CXCL1**	C-X-C motif chemokine ligand 1	3.254	0.001	0.031	Chemokine involved in neutrophil recruitment and activation [25]
**HLA-DRB6**	major histocompatibility complex, class II, DR beta 6 (pseudogene)	3.068	0.000	0.001	Component of the Major Histocompatibility Complex[26]
**HOXD11**	homeobox D11	-3.082	0.003	0.048	Critical in axial skeletal development and forelimb morphogenesis [27]
**PTPRQ**	protein tyrosine phosphatase receptor type Q	-3.259	0.000	0.009	phosphatase receptor involved in cellular proliferation and survival [28]
**AC011503.1**		-3.272	0.000	0.019	Uncharacterized protein
**KANK4**	KN motif and ankyrin repeat domains 4	-3.333	0.000	0.013	Control of cytoskeleton formation [29]
**FBP2**	fructose-bisphosphatase 2	-3.755	0.000	0.019	Regulator of cellular metabolic functions [30]
**C1orf158**	chromosome 1 open reading frame 158	-4.297	0.003	0.048	Uncharacterized protein

Note: log2Fold change demonstrates the expression of IFA + peptide compared to IFA alone. A positive Log2Fold change denotes higher expression in IFA + peptide compared to IFA alone. Negative Log2Fold change represents lower expression in IFA + peptide compared to IFA alone.

Supplementary Table 2. GSEA results showing significantly altered KEGG pathways ranked by the normalized enrichment score.

Pathway	Gene set size	Enrichment Score	Normalized Enrichment Score	p-Value	FDR-p Value
KEGG_GRAFT_VERSUS_HOST_DISEASE	37	0.783	2.553	0.000	0.000
KEGG_ANTIGEN_PROCESSING_AND_PRESENTATION	72	0.672	2.501	0.000	0.000
KEGG_CYTOKINE_CYTOKINE_RECEPTOR_INTERACTION	254	0.553	2.485	0.000	0.000
KEGG_ALLOGRAFT_REJECTION	35	0.765	2.464	0.000	0.000
KEGG_NATURAL_KILLER_CELL_MEDIATED_CYTOTOXICITY	124	0.602	2.443	0.000	0.000
KEGG_T_CELL_RECEPTOR_SIGNALING_PATHWAY	108	0.611	2.433	0.000	0.000
KEGG_INTESTINAL_IMMUNE_NETWORK_FOR_IGA_PRODUCTION	46	0.695	2.387	0.000	0.000
KEGG_TYPE_I_DIABETES_MELLITUS	41	0.708	2.369	0.000	0.000
KEGG_CHEMOKINE_SIGNALING_PATHWAY	187	0.543	2.354	0.000	0.000
KEGG_PRIMARY_IMMUNODEFICIENCY	35	0.719	2.314	0.000	0.000
KEGG_AUTOIMMUNE_THYROID_DISEASE	42	0.679	2.294	0.000	0.000
KEGG_CYTOSOLIC_DNA_SENSING_PATHWAY	45	0.630	2.145	0.000	0.000
KEGG_NOD_LIKE_RECEPTOR_SIGNALING_PATHWAY	62	0.579	2.111	0.000	0.000
KEGG_LEISHMANIA_INFECTION	70	0.550	2.039	0.000	0.000
KEGG_ASTHMA	28	0.671	2.036	0.000	0.001
KEGG_TOLL_LIKE_RECEPTOR_SIGNALING_PATHWAY	94	0.504	1.980	0.000	0.000
KEGG_SYSTEMIC_LUPUS_ERYTHEMATOSUS	55	0.554	1.964	0.000	0.001
KEGG_HEMATOPOIETIC_CELL_LINEAGE	84	0.514	1.963	0.000	0.000
KEGG_JAK_STAT_SIGNALING_PATHWAY	147	0.435	1.818	0.000	0.000
KEGG_CELL_ADHESION_MOLECULES_CAMS	130	0.414	1.700	0.000	0.002
KEGG_RIG_I_LIKE_RECEPTOR_SIGNALING_PATHWAY	62	0.462	1.685	0.004	0.022
KEGG_VIRAL_MYOCARDITIS	68	0.427	1.574	0.008	0.035
KEGG_CELL_CYCLE	124	0.373	1.514	0.003	0.017
KEGG_VASCULAR_SMOOTH_MUSCLE_CONTRACTION	112	-0.374	-1.455	0.010	0.041
KEGG_STARCH_AND_SUCROSE_METABOLISM	48	-0.454	-1.523	0.011	0.047
KEGG_MELANOMA	69	-0.438	-1.556	0.005	0.023
KEGG_DILATED_CARDIOMYOPATHY	89	-0.430	-1.612	0.003	0.017
KEGG_ADIPOCYTOKINE_SIGNALING_PATHWAY	67	-0.456	-1.615	0.005	0.023

KEGG_CITRATE_CYCLE_TCA_CYCLE	31	-0.532	-1.627	0.005	0.023
KEGG_GLYCEROLIPID_METABOLISM	49	-0.495	-1.665	0.004	0.019
KEGG_FATTY_ACID_METABOLISM	42	-0.512	-1.676	0.003	0.017
KEGG_NEUROACTIVE_LIGAND_RECEPTOR_INTERACTION	262	-0.391	-1.683	0.000	0.000
KEGG_PROXIMAL_TUBULE_BICARBONATE_RECLAMATION	23	-0.587	-1.685	0.005	0.023
KEGG_ABC_TRANSPORTERS	44	-0.512	-1.688	0.002	0.014
KEGG_INSULIN_SIGNALING_PATHWAY	136	-0.426	-1.700	0.000	0.001
KEGG_BUTANOATE_METABOLISM	34	-0.548	-1.709	0.005	0.023
KEGG_TERPENOID_BACKBONE_BIOSYNTHESIS	15	-0.667	-1.709	0.006	0.025
KEGG_PYRUVATE_METABOLISM	40	-0.539	-1.737	0.002	0.014
KEGG_PROPANOATE_METABOLISM	32	-0.574	-1.756	0.002	0.012
KEGG_VALINE_LEUCINE_AND_ISOLEUCINE_DEGRADATION	43	-0.544	-1.793	0.001	0.007
KEGG_BETA_ALANINE_METABOLISM	22	-0.654	-1.854	0.002	0.011
KEGG_BIOSYNTHESIS_OF_UNSATURATED_FATTY_ACIDS	22	-0.693	-1.964	0.000	0.002
KEGG_PEROXISOME	77	-0.548	-1.997	0.000	0.000
KEGG_PPAR_SIGNALING_PATHWAY	69	-0.593	-2.108	0.000	0.000

Supplementary Table 3. FDR-P values of all comparisons for each gene in figures 4A, 5A, 6A.

Gene	Condition 1	Condition 2	FDR-Pvalue	Significant?
CD80	Normal Skin	W1: IFA	1	ns
CD80	Normal Skin	W1: IFA + P	0.106535291	ns
CD80	Normal Skin	W1: IFA + pICLC + P	0.000768439	***
CD80	Normal Skin	W3 SSV: IFA	0.005815237	**
CD80	Normal Skin	W3 SSV: IFA + P	7.38E-05	***
CD80	Normal Skin	W3 SSV: IFA + pICLC + P	0.000146149	***
CD80	W1: IFA + P	W1: IFA	0.231588514	ns
CD80	W1: IFA + pICLC + P	W1: IFA + P	0.152111612	ns
CD80	W3 SSV: IFA	W1: IFA	0.04872854	*
CD80	W3 SSV: IFA + P	W1: IFA + P	0.010590248	*
CD80	W3 SSV: IFA + pICLC + P	W1: IFA + pICLC + P	0.619774613	ns
CD80	W3 SSV: IFA + P	W3 SSV: IFA	0.941353662	ns
CD80	W3 SSV: IFA + pICLC + P	W3 SSV: IFA	0.923543359	ns
CD80	W3 SSV: IFA + pICLC + P	W3 SSV: IFA + P	0.638361098	ns
CD83	Normal Skin	W1: IFA	1	ns
CD83	Normal Skin	W1: IFA + P	0.069224833	ns
CD83	Normal Skin	W1: IFA + pICLC + P	0.02196281	*
CD83	Normal Skin	W3 SSV: IFA	0.001869107	**
CD83	Normal Skin	W3 SSV: IFA + P	7.94E-05	***
CD83	Normal Skin	W3 SSV: IFA + pICLC + P	0.002446439	**
CD83	W1: IFA + P	W1: IFA	0.244706972	ns
CD83	W1: IFA + pICLC + P	W1: IFA + P	0.634647988	ns
CD83	W3 SSV: IFA	W1: IFA	0.042186175	*
CD83	W3 SSV: IFA + P	W1: IFA + P	0.076860159	ns
CD83	W3 SSV: IFA + pICLC + P	W1: IFA + pICLC + P	0.265528929	ns
CD83	W3 SSV: IFA + P	W3 SSV: IFA	0.998977672	ns
CD83	W3 SSV: IFA + pICLC + P	W3 SSV: IFA	0.441049819	ns
CD83	W3 SSV: IFA + pICLC + P	W3 SSV: IFA + P	0.184551264	ns
CD86	Normal Skin	W1: IFA	1	ns
CD86	Normal Skin	W1: IFA + P	0.165205475	ns
CD86	Normal Skin	W1: IFA + pICLC + P	0.002950351	**
CD86	Normal Skin	W3 SSV: IFA	0.001735111	**
CD86	Normal Skin	W3 SSV: IFA + P	2.45E-05	***
CD86	Normal Skin	W3 SSV: IFA + pICLC + P	0.000203032	***
CD86	W1: IFA + P	W1: IFA	0.550705427	ns
CD86	W1: IFA + pICLC + P	W1: IFA + P	0.373532483	ns
CD86	W3 SSV: IFA	W1: IFA	0.062841577	ns
CD86	W3 SSV: IFA + P	W1: IFA + P	0.00262272	**
CD86	W3 SSV: IFA + pICLC + P	W1: IFA + pICLC + P	0.277355555	ns
CD86	W3 SSV: IFA + P	W3 SSV: IFA	0.990939698	ns
CD86	W3 SSV: IFA + pICLC + P	W3 SSV: IFA	0.73645502	ns
CD86	W3 SSV: IFA + pICLC + P	W3 SSV: IFA + P	0.344927936	ns
CD40	Normal Skin	W1: IFA	1	ns
CD40	Normal Skin	W1: IFA + P	0.553507408	ns
CD40	Normal Skin	W1: IFA + pICLC + P	0.023166054	*
CD40	Normal Skin	W3 SSV: IFA	0.023862372	*
CD40	Normal Skin	W3 SSV: IFA + P	5.86E-05	***
CD40	Normal Skin	W3 SSV: IFA + pICLC + P	0.00388106	**
CD40	W1: IFA + P	W1: IFA	0.554824574	ns
CD40	W1: IFA + pICLC + P	W1: IFA + P	0.116414031	ns
CD40	W3 SSV: IFA	W1: IFA	0.059346237	ns
CD40	W3 SSV: IFA + P	W1: IFA + P	3.88E-05	***
CD40	W3 SSV: IFA + pICLC + P	W1: IFA + pICLC + P	0.387564291	ns
CD40	W3 SSV: IFA + P	W3 SSV: IFA	0.829148924	ns
CD40	W3 SSV: IFA + pICLC + P	W3 SSV: IFA	0.880313909	ns
CD40	W3 SSV: IFA + pICLC + P	W3 SSV: IFA + P	0.109091339	ns
CD40LG	Normal Skin	W1: IFA	1	ns
CD40LG	Normal Skin	W1: IFA + P	0.601705564	ns

CD40LG	Normal Skin	W1: IFA + pICLC + P	0.061051892	ns
CD40LG	Normal Skin	W3 SSV: IFA	0.002193346	**
CD40LG	Normal Skin	W3 SSV: IFA + P	0.000267538	***
CD40LG	Normal Skin	W3 SSV: IFA + pICLC + P	0.00118399	**
CD40LG	W1: IFA + P	W1: IFA	0.267198107	ns
CD40LG	W1: IFA + pICLC + P	W1: IFA + P	0.229621406	ns
CD40LG	W3 SSV: IFA	W1: IFA	0.001158955	**
CD40LG	W3 SSV: IFA + P	W1: IFA + P	0.000151042	***
CD40LG	W3 SSV: IFA + pICLC + P	W1: IFA + pICLC + P	0.013823312	*
CD40LG	W3 SSV: IFA + P	W3 SSV: IFA	0.995799259	ns
CD40LG	W3 SSV: IFA + pICLC + P	W3 SSV: IFA	0.577380166	ns
CD40LG	W3 SSV: IFA + pICLC + P	W3 SSV: IFA + P	0.486789341	ns
BATF3	Normal Skin	W1: IFA	1	ns
BATF3	Normal Skin	W1: IFA + P	0.234285493	ns
BATF3	Normal Skin	W1: IFA + pICLC + P	0.001371035	**
BATF3	Normal Skin	W3 SSV: IFA	0.001869107	**
BATF3	Normal Skin	W3 SSV: IFA + P	0.000104572	***
BATF3	Normal Skin	W3 SSV: IFA + pICLC + P	0.000303517	***
BATF3	W1: IFA + P	W1: IFA	0.520880305	ns
BATF3	W1: IFA + pICLC + P	W1: IFA + P	0.098248933	ns
BATF3	W3 SSV: IFA	W1: IFA	0.038049266	*
BATF3	W3 SSV: IFA + P	W1: IFA + P	0.004230693	**
BATF3	W3 SSV: IFA + pICLC + P	W1: IFA + pICLC + P	0.656352271	ns
BATF3	W3 SSV: IFA + P	W3 SSV: IFA	0.999844543	ns
BATF3	W3 SSV: IFA + pICLC + P	W3 SSV: IFA	0.726019284	ns
BATF3	W3 SSV: IFA + pICLC + P	W3 SSV: IFA + P	0.548076044	ns
CD4	Normal Skin	W1: IFA	1	ns
CD4	Normal Skin	W1: IFA + P	0.403761152	ns
CD4	Normal Skin	W1: IFA + pICLC + P	0.053573387	ns
CD4	Normal Skin	W3 SSV: IFA	0.001991315	**
CD4	Normal Skin	W3 SSV: IFA + P	8.60E-05	***
CD4	Normal Skin	W3 SSV: IFA + pICLC + P	0.000796859	***
CD4	W1: IFA + P	W1: IFA	0.633969851	ns
CD4	W1: IFA + pICLC + P	W1: IFA + P	0.586632802	ns
CD4	W3 SSV: IFA	W1: IFA	0.02516306	*
CD4	W3 SSV: IFA + P	W1: IFA + P	0.000452937	***
CD4	W3 SSV: IFA + pICLC + P	W1: IFA + pICLC + P	0.011781842	*
CD4	W3 SSV: IFA + P	W3 SSV: IFA	0.998977672	ns
CD4	W3 SSV: IFA + pICLC + P	W3 SSV: IFA	0.604108813	ns
CD4	W3 SSV: IFA + pICLC + P	W3 SSV: IFA + P	0.349334428	ns
CD8A	Normal Skin	W1: IFA	1	ns
CD8A	Normal Skin	W1: IFA + P	0.085492699	ns
CD8A	Normal Skin	W1: IFA + pICLC + P	0.000142329	***
CD8A	Normal Skin	W3 SSV: IFA	0.006445787	**
CD8A	Normal Skin	W3 SSV: IFA + P	2.29E-05	***
CD8A	Normal Skin	W3 SSV: IFA + pICLC + P	5.73E-07	***
CD8A	W1: IFA + P	W1: IFA	0.175588343	ns
CD8A	W1: IFA + pICLC + P	W1: IFA + P	0.1563349	ns
CD8A	W3 SSV: IFA	W1: IFA	0.043452503	*
CD8A	W3 SSV: IFA + P	W1: IFA + P	0.012581361	*
CD8A	W3 SSV: IFA + pICLC + P	W1: IFA + pICLC + P	0.024870915	*
CD8A	W3 SSV: IFA + P	W3 SSV: IFA	0.91669961	ns
CD8A	W3 SSV: IFA + pICLC + P	W3 SSV: IFA	0.448869728	ns
CD8A	W3 SSV: IFA + pICLC + P	W3 SSV: IFA + P	0.832672283	ns
IFNG	Normal Skin	W1: IFA	1	ns
IFNG	Normal Skin	W1: IFA + P	0.277994863	ns
IFNG	Normal Skin	W1: IFA + pICLC + P	0.004569814	**
IFNG	Normal Skin	W3 SSV: IFA	0.022563359	*
IFNG	Normal Skin	W3 SSV: IFA + P	7.86E-05	***
IFNG	Normal Skin	W3 SSV: IFA + pICLC + P	0.000236121	***
IFNG	W1: IFA + P	W1: IFA	0.244614267	ns
IFNG	W1: IFA + pICLC + P	W1: IFA + P	0.051337583	ns
IFNG	W3 SSV: IFA	W1: IFA	0.039430886	*
IFNG	W3 SSV: IFA + P	W1: IFA + P	0.000127689	***
IFNG	W3 SSV: IFA + pICLC + P	W1: IFA + pICLC + P	0.092644244	ns
IFNG	W3 SSV: IFA + P	W3 SSV: IFA	0.688519877	ns
IFNG	W3 SSV: IFA + pICLC + P	W3 SSV: IFA	0.616748064	ns
IFNG	W3 SSV: IFA + pICLC + P	W3 SSV: IFA + P	0.54130969	ns
TBX21	Normal Skin	W1: IFA	1	ns
TBX21	Normal Skin	W1: IFA + P	0.301391253	ns
TBX21	Normal Skin	W1: IFA + pICLC + P	0.005878372	**
TBX21	Normal Skin	W3 SSV: IFA	0.011508377	*
TBX21	Normal Skin	W3 SSV: IFA + P	0.000168162	***
TBX21	Normal Skin	W3 SSV: IFA + pICLC + P	6.13E-05	***
TBX21	W1: IFA + P	W1: IFA	0.183407086	ns
TBX21	W1: IFA + pICLC + P	W1: IFA + P	0.194663021	ns
TBX21	W3 SSV: IFA	W1: IFA	0.012836676	*
TBX21	W3 SSV: IFA + P	W1: IFA + P	0.003161057	**
TBX21	W3 SSV: IFA + pICLC + P	W1: IFA + pICLC + P	0.025078223	*
TBX21	W3 SSV: IFA + P	W3 SSV: IFA	0.966088604	ns
TBX21	W3 SSV: IFA + pICLC + P	W3 SSV: IFA	0.810879311	ns
TBX21	W3 SSV: IFA + pICLC + P	W3 SSV: IFA + P	0.91607758	ns
STAT1	Normal Skin	W1: IFA	1	ns
STAT1	Normal Skin	W1: IFA + P	0.142912332	ns

STAT1	Normal Skin	W1: IFA + pICLC + P	5.99E-05	***
STAT1	Normal Skin	W3 SSV: IFA	0.061022511	ns
STAT1	Normal Skin	W3 SSV: IFA + P	2.51E-05	***
STAT1	Normal Skin	W3 SSV: IFA + pICLC + P	7.52E-07	***
STAT1	W1: IFA + P	W1: IFA	0.520053952	ns
STAT1	W1: IFA + pICLC + P	W1: IFA + P	0.029554687	*
STAT1	W3 SSV: IFA	W1: IFA	0.450489753	ns
STAT1	W3 SSV: IFA + P	W1: IFA + P	0.004498665	**
STAT1	W3 SSV: IFA + pICLC + P	W1: IFA + pICLC + P	0.096645573	ns
STAT1	W3 SSV: IFA + P	W3 SSV: IFA	0.527496179	ns
STAT1	W3 SSV: IFA + pICLC + P	W3 SSV: IFA	0.109138181	ns
STAT1	W3 SSV: IFA + pICLC + P	W3 SSV: IFA + P	0.869270626	ns
GATA3	Normal Skin	W1: IFA	1	ns
GATA3	Normal Skin	W1: IFA + P	0.73909022	ns
GATA3	Normal Skin	W1: IFA + pICLC + P	0.479410923	ns
GATA3	Normal Skin	W3 SSV: IFA	0.586325493	ns
GATA3	Normal Skin	W3 SSV: IFA + P	0.803762557	ns
GATA3	Normal Skin	W3 SSV: IFA + pICLC + P	0.975572992	ns
GATA3	W1: IFA + P	W1: IFA	0.405204242	ns
GATA3	W1: IFA + pICLC + P	W1: IFA + P	0.936887135	ns
GATA3	W3 SSV: IFA	W1: IFA	0.80946911	ns
GATA3	W3 SSV: IFA + P	W1: IFA + P	0.107568945	ns
GATA3	W3 SSV: IFA + pICLC + P	W1: IFA + pICLC + P	0.028424853	*
GATA3	W3 SSV: IFA + P	W3 SSV: IFA	0.955093581	ns
GATA3	W3 SSV: IFA + pICLC + P	W3 SSV: IFA	0.5337106	ns
GATA3	W3 SSV: IFA + pICLC + P	W3 SSV: IFA + P	0.795269195	ns
RORC	Normal Skin	W1: IFA	1	ns
RORC	Normal Skin	W1: IFA + P	0.992513356	ns
RORC	Normal Skin	W1: IFA + pICLC + P	0.96632296	ns
RORC	Normal Skin	W3 SSV: IFA	0.208635568	ns
RORC	Normal Skin	W3 SSV: IFA + P	0.049876996	*
RORC	Normal Skin	W3 SSV: IFA + pICLC + P	0.272221073	ns
RORC	W1: IFA + P	W1: IFA	0.886731047	ns
RORC	W1: IFA + pICLC + P	W1: IFA + P	0.976221081	ns
RORC	W3 SSV: IFA	W1: IFA	0.302736994	ns
RORC	W3 SSV: IFA + P	W1: IFA + P	0.002153749	**
RORC	W3 SSV: IFA + pICLC + P	W1: IFA + pICLC + P	0.003955918	**
RORC	W3 SSV: IFA + P	W3 SSV: IFA	0.995799259	ns
RORC	W3 SSV: IFA + pICLC + P	W3 SSV: IFA	0.618677894	ns
RORC	W3 SSV: IFA + pICLC + P	W3 SSV: IFA + P	0.308574734	ns
FOXP3	Normal Skin	W1: IFA	1	ns
FOXP3	Normal Skin	W1: IFA + P	0.684830049	ns
FOXP3	Normal Skin	W1: IFA + pICLC + P	0.190435173	ns
FOXP3	Normal Skin	W3 SSV: IFA	0.042662165	*
FOXP3	Normal Skin	W3 SSV: IFA + P	0.002190521	**
FOXP3	Normal Skin	W3 SSV: IFA + pICLC + P	0.008059791	**
FOXP3	W1: IFA + P	W1: IFA	0.431026986	ns
FOXP3	W1: IFA + pICLC + P	W1: IFA + P	0.512270784	ns
FOXP3	W3 SSV: IFA	W1: IFA	0.031087307	*
FOXP3	W3 SSV: IFA + P	W1: IFA + P	0.001293996	**
FOXP3	W3 SSV: IFA + pICLC + P	W1: IFA + pICLC + P	0.016042083	*
FOXP3	W3 SSV: IFA + P	W3 SSV: IFA	0.986607086	ns
FOXP3	W3 SSV: IFA + pICLC + P	W3 SSV: IFA	0.895664995	ns
FOXP3	W3 SSV: IFA + pICLC + P	W3 SSV: IFA + P	0.523473556	ns
PD1	Normal Skin	W1: IFA	1	ns
PD1	Normal Skin	W1: IFA + P	0.417136626	ns
PD1	Normal Skin	W1: IFA + pICLC + P	0.001523664	**
PD1	Normal Skin	W3 SSV: IFA	0.007233044	**
PD1	Normal Skin	W3 SSV: IFA + P	2.19E-05	***
PD1	Normal Skin	W3 SSV: IFA + pICLC + P	0.000246404	***
PD1	W1: IFA + P	W1: IFA	0.531791551	ns
PD1	W1: IFA + pICLC + P	W1: IFA + P	0.016049108	*
PD1	W3 SSV: IFA	W1: IFA	0.039647902	*
PD1	W3 SSV: IFA + P	W1: IFA + P	5.16E-05	***
PD1	W3 SSV: IFA + pICLC + P	W1: IFA + pICLC + P	0.54790876	ns
PD1	W3 SSV: IFA + P	W3 SSV: IFA	0.903688722	ns
PD1	W3 SSV: IFA + pICLC + P	W3 SSV: IFA	0.970426286	ns
PD1	W3 SSV: IFA + pICLC + P	W3 SSV: IFA + P	0.29639221	ns
LAG3	Normal Skin	W1: IFA	1	ns
LAG3	Normal Skin	W1: IFA + P	0.378958845	ns
LAG3	Normal Skin	W1: IFA + pICLC + P	0.001876713	**
LAG3	Normal Skin	W3 SSV: IFA	0.045994878	*
LAG3	Normal Skin	W3 SSV: IFA + P	0.00016211	***
LAG3	Normal Skin	W3 SSV: IFA + pICLC + P	2.24E-06	***
LAG3	W1: IFA + P	W1: IFA	0.367307225	ns
LAG3	W1: IFA + pICLC + P	W1: IFA + P	0.029297214	*
LAG3	W3 SSV: IFA	W1: IFA	0.088954818	ns
LAG3	W3 SSV: IFA + P	W1: IFA + P	0.001244695	**
LAG3	W3 SSV: IFA + pICLC + P	W1: IFA + pICLC + P	0.002136856	**
LAG3	W3 SSV: IFA + P	W3 SSV: IFA	0.821989549	ns
LAG3	W3 SSV: IFA + pICLC + P	W3 SSV: IFA	0.19003287	ns
LAG3	W3 SSV: IFA + pICLC + P	W3 SSV: IFA + P	0.630303093	ns
TIM-3	Normal Skin	W1: IFA	1	ns
TIM-3	Normal Skin	W1: IFA + P	0.296067639	ns

TIM-3	Normal Skin	W1: IFA + pICLC + P	0.056983315	ns
TIM-3	Normal Skin	W3 SSV: IFA	0.009789901	**
TIM-3	Normal Skin	W3 SSV: IFA + P	0.000449181	***
TIM-3	Normal Skin	W3 SSV: IFA + pICLC + P	0.003616052	**
TIM-3	W1: IFA + P	W1: IFA	0.740817012	ns
TIM-3	W1: IFA + pICLC + P	W1: IFA + P	0.870799842	ns
TIM-3	W3 SSV: IFA	W1: IFA	0.180074194	ns
TIM-3	W3 SSV: IFA + P	W1: IFA + P	0.011673532	*
TIM-3	W3 SSV: IFA + pICLC + P	W1: IFA + pICLC + P	0.098021208	ns
TIM-3	W3 SSV: IFA + P	W3 SSV: IFA	0.994098514	ns
TIM-3	W3 SSV: IFA + pICLC + P	W3 SSV: IFA	0.70755146	ns
TIM-3	W3 SSV: IFA + pICLC + P	W3 SSV: IFA + P	0.377071734	ns
TICAM1	Normal Skin	W1: IFA	1	ns
TICAM1	Normal Skin	W1: IFA + P	0.795634732	ns
TICAM1	Normal Skin	W1: IFA + pICLC + P	0.058460425	ns
TICAM1	Normal Skin	W3 SSV: IFA	0.41854247	ns
TICAM1	Normal Skin	W3 SSV: IFA + P	0.033886014	*
TICAM1	Normal Skin	W3 SSV: IFA + pICLC + P	0.000858657	***
TICAM1	W1: IFA + P	W1: IFA	0.2043692	ns
TICAM1	W1: IFA + pICLC + P	W1: IFA + P	0.049792283	*
TICAM1	W3 SSV: IFA	W1: IFA	0.083435612	ns
TICAM1	W3 SSV: IFA + P	W1: IFA + P	0.023109022	*
TICAM1	W3 SSV: IFA + pICLC + P	W1: IFA + pICLC + P	0.010050422	*
TICAM1	W3 SSV: IFA + P	W3 SSV: IFA	0.894740887	ns
TICAM1	W3 SSV: IFA + pICLC + P	W3 SSV: IFA	0.147981415	ns
TICAM1	W3 SSV: IFA + pICLC + P	W3 SSV: IFA + P	0.381479518	ns
MYD88	Normal Skin	W1: IFA	1	ns
MYD88	Normal Skin	W1: IFA + P	0.324666971	ns
MYD88	Normal Skin	W1: IFA + pICLC + P	0.004531038	**
MYD88	Normal Skin	W3 SSV: IFA	0.042074387	*
MYD88	Normal Skin	W3 SSV: IFA + P	0.000379406	***
MYD88	Normal Skin	W3 SSV: IFA + pICLC + P	9.63E-05	***
MYD88	W1: IFA + P	W1: IFA	0.53622382	ns
MYD88	W1: IFA + pICLC + P	W1: IFA + P	0.138386014	ns
MYD88	W3 SSV: IFA	W1: IFA	0.193799974	ns
MYD88	W3 SSV: IFA + P	W1: IFA + P	0.00692755	**
MYD88	W3 SSV: IFA + pICLC + P	W1: IFA + pICLC + P	0.073919006	ns
MYD88	W3 SSV: IFA + P	W3 SSV: IFA	0.919814797	ns
MYD88	W3 SSV: IFA + pICLC + P	W3 SSV: IFA	0.616617969	ns
MYD88	W3 SSV: IFA + pICLC + P	W3 SSV: IFA + P	0.97826591	ns
ARG1	Normal Skin	W1: IFA	1	ns
ARG1	Normal Skin	W1: IFA + P	0.748130031	ns
ARG1	Normal Skin	W1: IFA + pICLC + P	0.464873931	ns
ARG1	Normal Skin	W3 SSV: IFA	0.763867727	ns
ARG1	Normal Skin	W3 SSV: IFA + P	0.589485331	ns
ARG1	Normal Skin	W3 SSV: IFA + pICLC + P	0.851116364	ns
ARG1	W1: IFA + P	W1: IFA	0.644129972	ns
ARG1	W1: IFA + pICLC + P	W1: IFA + P	0.907322252	ns
ARG1	W3 SSV: IFA	W1: IFA	0.764436026	ns
ARG1	W3 SSV: IFA + P	W1: IFA + P	0.035345336	*
ARG1	W3 SSV: IFA + pICLC + P	W1: IFA + pICLC + P	0.006218425	**
ARG1	W3 SSV: IFA + P	W3 SSV: IFA	0.997801558	ns
ARG1	W3 SSV: IFA + pICLC + P	W3 SSV: IFA	0.859927178	ns
ARG1	W3 SSV: IFA + pICLC + P	W3 SSV: IFA + P	0.689322195	ns

Supplementary Table 4. FDR-P values of all comparisons for each gene in figure's 4B, 5B, 6B.

Gene	Condition 1	Condition 2	FDR-Pvalue	Significant?
CD80	Normal Skin	W1: IFA + P	0.106535291	ns
CD80	Normal Skin	W3 SSV: IFA + P	7.38E-05	***
CD80	Normal Skin	W3 RV: IFA + P	0.76443872	ns
CD80	W3 SSV: IFA + P	W3 RV: IFA + P	0.000223518	***
CD80	W1: IFA + P	W3 RV: IFA + P	0.910136267	ns
CD80	Normal Skin	W3 RV: IFA + pICLC + P	0.033049609	*
CD80	W1: IFA + pICLC + P	W3 RV: IFA + pICLC + P	0.910136267	ns
CD80	W3 SSV: IFA + pICLC + P	W3 RV: IFA + pICLC + P	0.288646884	ns
CD83	Normal Skin	W1: IFA + P	0.069224833	ns
CD83	Normal Skin	W3 SSV: IFA + P	7.94E-05	***
CD83	Normal Skin	W3 RV: IFA + P	0.980134489	ns
CD83	W3 SSV: IFA + P	W3 RV: IFA + P	5.02E-05	***
CD83	W1: IFA + P	W3 RV: IFA + P	0.905419821	ns
CD83	Normal Skin	W3 RV: IFA + pICLC + P	0.27912594	ns
CD83	W1: IFA + pICLC + P	W3 RV: IFA + pICLC + P	0.905419821	ns
CD83	W3 SSV: IFA + pICLC + P	W3 RV: IFA + pICLC + P	0.147940092	ns
CD86	Normal Skin	W1: IFA + P	0.165205475	ns
CD86	Normal Skin	W3 SSV: IFA + P	2.45E-05	***
CD86	Normal Skin	W3 RV: IFA + P	0.886201324	ns
CD86	W3 SSV: IFA + P	W3 RV: IFA + P	7.24E-05	***
CD86	W1: IFA + P	W3 RV: IFA + P	0.967903317	ns
CD86	Normal Skin	W3 RV: IFA + pICLC + P	0.056779238	ns
CD86	W1: IFA + pICLC + P	W3 RV: IFA + pICLC + P	0.967903317	ns

CD86	W3 SSV: IFA + pICLC + P	W3 RV: IFA + pICLC + P	0.347204194	ns
CD40	Normal Skin	W1: IFA + P	0.553507408	ns
CD40	Normal Skin	W3 SSV: IFA + P	5.86E-05	***
CD40	Normal Skin	W3 RV: IFA + P	0.737557226	ns
CD40	W3 SSV: IFA + P	W3 RV: IFA + P	0.000674476	***
CD40	W1: IFA + P	W3 RV: IFA + P	0.922238713	ns
CD40	Normal Skin	W3 RV: IFA + pICLC + P	0.23882714	ns
CD40	W1: IFA + pICLC + P	W3 RV: IFA + pICLC + P	0.922238713	ns
CD40	W3 SSV: IFA + pICLC + P	W3 RV: IFA + pICLC + P	0.254788139	ns
CD40LG	Normal Skin	W1: IFA + P	0.601705564	ns
CD40LG	Normal Skin	W3 SSV: IFA + P	0.000267538	***
CD40LG	Normal Skin	W3 RV: IFA + P	0.85733342	ns
CD40LG	W3 SSV: IFA + P	W3 RV: IFA + P	5.42E-05	***
CD40LG	W1: IFA + P	W3 RV: IFA + P	0.995444309	ns
CD40LG	Normal Skin	W3 RV: IFA + pICLC + P	0.204234182	ns
CD40LG	W1: IFA + pICLC + P	W3 RV: IFA + pICLC + P	0.995444309	ns
CD40LG	W3 SSV: IFA + pICLC + P	W3 RV: IFA + pICLC + P	0.149078054	ns
BATF3	Normal Skin	W1: IFA + P	0.234285493	ns
BATF3	Normal Skin	W3 SSV: IFA + P	0.000104572	***
BATF3	Normal Skin	W3 RV: IFA + P	0.825916783	ns
BATF3	W3 SSV: IFA + P	W3 RV: IFA + P	0.000486573	***
BATF3	W1: IFA + P	W3 RV: IFA + P	0.894014538	ns
BATF3	Normal Skin	W3 RV: IFA + pICLC + P	0.218123585	ns
BATF3	W1: IFA + pICLC + P	W3 RV: IFA + pICLC + P	0.894014538	ns
BATF3	W3 SSV: IFA + pICLC + P	W3 RV: IFA + pICLC + P	0.056421643	ns
CD4	Normal Skin	W1: IFA + P	0.403761152	ns
CD4	Normal Skin	W3 SSV: IFA + P	8.60E-05	***
CD4	Normal Skin	W3 RV: IFA + P	0.928266469	ns
CD4	W3 SSV: IFA + P	W3 RV: IFA + P	2.98E-05	***
CD4	W1: IFA + P	W3 RV: IFA + P	0.984257495	ns
CD4	Normal Skin	W3 RV: IFA + pICLC + P	0.217666049	ns
CD4	W1: IFA + pICLC + P	W3 RV: IFA + pICLC + P	0.984257495	ns
CD4	W3 SSV: IFA + pICLC + P	W3 RV: IFA + pICLC + P	0.11482172	ns
CD8A	Normal Skin	W1: IFA + P	0.085492699	ns
CD8A	Normal Skin	W3 SSV: IFA + P	2.29E-05	***
CD8A	Normal Skin	W3 RV: IFA + P	0.720829789	ns
CD8A	W3 SSV: IFA + P	W3 RV: IFA + P	0.000299514	***
CD8A	W1: IFA + P	W3 RV: IFA + P	0.950732209	ns
CD8A	Normal Skin	W3 RV: IFA + pICLC + P	0.01537181	*
CD8A	W1: IFA + pICLC + P	W3 RV: IFA + pICLC + P	0.950732209	ns
CD8A	W3 SSV: IFA + pICLC + P	W3 RV: IFA + pICLC + P	0.090957415	ns
IFNG	Normal Skin	W1: IFA + P	0.277994863	ns
IFNG	Normal Skin	W3 SSV: IFA + P	7.86E-05	***
IFNG	Normal Skin	W3 RV: IFA + P	0.882663938	ns
IFNG	W3 SSV: IFA + P	W3 RV: IFA + P	3.79E-05	***
IFNG	W1: IFA + P	W3 RV: IFA + P	0.983188954	ns
IFNG	Normal Skin	W3 RV: IFA + pICLC + P	0.036430218	*
IFNG	W1: IFA + pICLC + P	W3 RV: IFA + pICLC + P	0.983188954	ns
IFNG	W3 SSV: IFA + pICLC + P	W3 RV: IFA + pICLC + P	0.261670608	ns
TBX21	Normal Skin	W1: IFA + P	0.301391253	ns
TBX21	Normal Skin	W3 SSV: IFA + P	0.000168162	***
TBX21	Normal Skin	W3 RV: IFA + P	0.539257063	ns
TBX21	W3 SSV: IFA + P	W3 RV: IFA + P	3.38E-06	***
TBX21	W1: IFA + P	W3 RV: IFA + P	0.988235511	ns
TBX21	Normal Skin	W3 RV: IFA + pICLC + P	0.059377385	ns
TBX21	W1: IFA + pICLC + P	W3 RV: IFA + pICLC + P	0.988235511	ns
TBX21	W3 SSV: IFA + pICLC + P	W3 RV: IFA + pICLC + P	0.168049843	ns
STAT1	Normal Skin	W1: IFA + P	0.142912332	ns
STAT1	Normal Skin	W3 SSV: IFA + P	2.51E-05	***
STAT1	Normal Skin	W3 RV: IFA + P	0.508457256	ns
STAT1	W3 SSV: IFA + P	W3 RV: IFA + P	0.002094592	**
STAT1	W1: IFA + P	W3 RV: IFA + P	0.899929076	ns
STAT1	Normal Skin	W3 RV: IFA + pICLC + P	0.034653928	*
STAT1	W1: IFA + pICLC + P	W3 RV: IFA + pICLC + P	0.899929076	ns
STAT1	W3 SSV: IFA + pICLC + P	W3 RV: IFA + pICLC + P	0.042864044	*
GATA3	Normal Skin	W1: IFA + P	0.73909022	ns
GATA3	Normal Skin	W3 SSV: IFA + P	0.803762557	ns
GATA3	Normal Skin	W3 RV: IFA + P	0.298683975	ns
GATA3	W3 SSV: IFA + P	W3 RV: IFA + P	0.041330023	*
GATA3	W1: IFA + P	W3 RV: IFA + P	0.968986563	ns
GATA3	Normal Skin	W3 RV: IFA + pICLC + P	0.523893247	ns
GATA3	W1: IFA + pICLC + P	W3 RV: IFA + pICLC + P	0.968986563	ns
GATA3	W3 SSV: IFA + pICLC + P	W3 RV: IFA + pICLC + P	0.12324254	ns
RORC	Normal Skin	W1: IFA + P	0.992513356	ns
RORC	Normal Skin	W3 SSV: IFA + P	0.049876996	*
RORC	Normal Skin	W3 RV: IFA + P	0.801701037	ns
RORC	W3 SSV: IFA + P	W3 RV: IFA + P	0.008996416	**
RORC	W1: IFA + P	W3 RV: IFA + P	0.982614639	ns
RORC	Normal Skin	W3 RV: IFA + pICLC + P	0.922562725	ns
RORC	W1: IFA + pICLC + P	W3 RV: IFA + pICLC + P	0.982614639	ns
RORC	W3 SSV: IFA + pICLC + P	W3 RV: IFA + pICLC + P	0.074897662	ns
FOXP3	Normal Skin	W1: IFA + P	0.684830049	ns
FOXP3	Normal Skin	W3 SSV: IFA + P	0.002190521	**
FOXP3	Normal Skin	W3 RV: IFA + P	0.963330726	ns

FOXP3	W3 SSV: IFA + P	W3 RV: IFA + P	0.002662953	**
FOXP3	W1: IFA + P	W3 RV: IFA + P	0.972566004	ns
FOXP3	Normal Skin	W3 RV: IFA + pICLC + P	0.290594995	ns
FOXP3	W1: IFA + pICLC + P	W3 RV: IFA + pICLC + P	0.972566004	ns
FOXP3	W3 SSV: IFA + pICLC + P	W3 RV: IFA + pICLC + P	0.290178454	ns
PD1	Normal Skin	W1: IFA + P	0.417136626	ns
PD1	Normal Skin	W3 SSV: IFA + P	2.19E-05	***
PD1	Normal Skin	W3 RV: IFA + P	0.892728444	ns
PD1	W3 SSV: IFA + P	W3 RV: IFA + P	5.70E-06	***
PD1	W1: IFA + P	W3 RV: IFA + P	0.951552029	ns
PD1	Normal Skin	W3 RV: IFA + pICLC + P	0.047816078	*
PD1	W1: IFA + pICLC + P	W3 RV: IFA + pICLC + P	0.951552029	ns
PD1	W3 SSV: IFA + pICLC + P	W3 RV: IFA + pICLC + P	0.439701177	ns
LAG3	Normal Skin	W1: IFA + P	0.378958845	ns
LAG3	Normal Skin	W3 SSV: IFA + P	0.00016211	***
LAG3	Normal Skin	W3 RV: IFA + P	0.795940658	ns
LAG3	W3 SSV: IFA + P	W3 RV: IFA + P	0.001018373	**
LAG3	W1: IFA + P	W3 RV: IFA + P	0.910136267	ns
LAG3	Normal Skin	W3 RV: IFA + pICLC + P	0.005443709	**
LAG3	W1: IFA + pICLC + P	W3 RV: IFA + pICLC + P	0.910136267	ns
LAG3	W3 SSV: IFA + pICLC + P	W3 RV: IFA + pICLC + P	0.363870747	ns
TIM-3	Normal Skin	W1: IFA + P	0.296067639	ns
TIM-3	Normal Skin	W3 SSV: IFA + P	0.000449181	***
TIM-3	Normal Skin	W3 RV: IFA + P	0.755142808	ns
TIM-3	W3 SSV: IFA + P	W3 RV: IFA + P	2.98E-05	***
TIM-3	W1: IFA + P	W3 RV: IFA + P	0.958873477	ns
TIM-3	Normal Skin	W3 RV: IFA + pICLC + P	0.282087544	ns
TIM-3	W1: IFA + pICLC + P	W3 RV: IFA + pICLC + P	0.958873477	ns
TIM-3	W3 SSV: IFA + pICLC + P	W3 RV: IFA + pICLC + P	0.187346139	ns
TICAM1	Normal Skin	W1: IFA + P	0.795634732	ns
TICAM1	Normal Skin	W3 SSV: IFA + P	0.033886014	*
TICAM1	Normal Skin	W3 RV: IFA + P	0.710255573	ns
TICAM1	W3 SSV: IFA + P	W3 RV: IFA + P	0.147425086	ns
TICAM1	W1: IFA + P	W3 RV: IFA + P	0.894014538	ns
TICAM1	Normal Skin	W3 RV: IFA + pICLC + P	0.720759063	ns
TICAM1	W1: IFA + pICLC + P	W3 RV: IFA + pICLC + P	0.894014538	ns
TICAM1	W3 SSV: IFA + pICLC + P	W3 RV: IFA + pICLC + P	0.00393303	**
MYD88	Normal Skin	W1: IFA + P	0.324666971	ns
MYD88	Normal Skin	W3 SSV: IFA + P	0.000379406	***
MYD88	Normal Skin	W3 RV: IFA + P	0.712236078	ns
MYD88	W3 SSV: IFA + P	W3 RV: IFA + P	0.004206942	**
MYD88	W1: IFA + P	W3 RV: IFA + P	0.899929076	ns
MYD88	Normal Skin	W3 RV: IFA + pICLC + P	0.190065818	ns
MYD88	W1: IFA + pICLC + P	W3 RV: IFA + pICLC + P	0.899929076	ns
MYD88	W3 SSV: IFA + pICLC + P	W3 RV: IFA + pICLC + P	0.042742622	*
ARG1	Normal Skin	W1: IFA + P	0.748130031	ns
ARG1	Normal Skin	W3 SSV: IFA + P	0.589485331	ns
ARG1	Normal Skin	W3 RV: IFA + P	0.391870798	ns
ARG1	W3 SSV: IFA + P	W3 RV: IFA + P	0.033776356	*
ARG1	W1: IFA + P	W3 RV: IFA + P	0.99247624	ns
ARG1	Normal Skin	W3 RV: IFA + pICLC + P	0.590473867	ns
ARG1	W1: IFA + pICLC + P	W3 RV: IFA + pICLC + P	0.99247624	ns
ARG1	W3 SSV: IFA + pICLC + P	W3 RV: IFA + pICLC + P	0.107934272	ns

SUPPLEMENTARY METHODS

Clinical Trial Methods

Mel 48: For Mel48, participants were immunized with MELITAC 12.1 (100µg of each of 12 melanoma peptides recognized by CD8 T cells (12MP) + 200µg of tetanus toxoid helper peptide (Tet))[1] emulsified in IFA (Montanide ISA-51 VG, Seppic) in one skin site on days 1, 8, 15, 29, 36, and 43. An additional sequence of vaccines were administered at one site on a different extremity to induce a similar VSME, solely for the purpose of VSME biopsies.

Participants were randomized to have the VSME biopsies a week after 1 or more vaccines. These “replicate” vaccines contained either IFA only (group 1) or IFA + MELITAC 12.1 (group 2). The replicate vaccines were administered until the date of the VSME biopsy. For the present study, VSME biopsies of the replicate site were obtained one week after the first vaccine (week 1, groups 1B/2B) or one week after the third vaccine (week 3, groups 1C/2C). Vaccine sites were marked using felt-tip pen in order to identify them at subsequent weeks for same-site vaccination. Normal skin controls were obtained pre-treatment for a subset of patients. Details of these methods have been reported.[2]

Mel 58: For the Mel58 trial, participants were also vaccinated with MELITAC12.1, and for the samples used in this analysis, the adjuvant preparation consisted of the TLR agonist polyICLC (pICLC, Hiltonol, Oncovir, Washington, DC, USA) emulsified with IFA, all collected one week after the first vaccine (week 1, groups 2B/2C). Details of these methods have been reported.[3]

Mel 60: On the Mel60 trial, participants were immunized with 7 long melanoma peptides (29-31 amino acids each), each containing a minimal epitope for CD8 T cells (LPV7[4]) (300µg per dose), with or without Tet helper peptide (200 µg per dose) with IFA and/or pICLC. The study included 7 adjuvant combinations: those analyzed for the present study included peptides with IFA alone or IFA + pICLC. Participants were vaccinated on days 1, 8, 15, 36, 57, and 78, at different extremity skin sites for each vaccine (rotating-site vaccines, RV) for groups A (IFA + LPV7+Tet) and E (IFA + pICLC+LPV7+Tet) or at the same site each week (same-site vaccination, SSV) for group E2 (IFA + pICLC+LPV7+Tet) For the present analysis, vaccine-site biopsy specimens were obtained one week after vaccine 1 (day 8, week 1) or one week after vaccine 3 (day 22, week 3) after RV or SSV. Biopsies on week 3 were taken at the site of the most recent vaccine.

Mel 63: On the Mel63 trial, participants were immunized with 6 Class II MHC restricted melanoma peptides (6MHP)[5 6] emulsified either in IFA alone or in IFA + pICLC, with vaccines injected in the same extremity skin site (SSV) on days 1, 8, and 15. Punch biopsy specimens from 1 week after the first vaccine (week 1) and 1 week after the third vaccine (week 3) were used in the present analysis.

<u>Tissue collection, RNA processing, and library preparation</u>

RNA was extracted from the RNAlater stored samples as previously described,[7] using the RNeasy Lipid Tissue MiniKit (Qiagen, Germantown, MD, USA) according to the manufacturer’s specifications. The RNA was processed for library prep as previously described.[7] The UVA School of Medicine’s Genome Analysis and Technology Core (GATC) performed the library preparation and final purified libraries underwent quantification and sizing using the Invitrogen

Qubit 3 Fluorometer (ThermoFisher Scientific Inc.,Waltham, MA) and Agilent Technologies 4200 TapeStation (Agilent Technologies Inc., Santa Clara, CA)

Statistical Analysis

The count table was imported to R for differential gene expression analysis. Low expressed genes (genes expressed only in few samples with low counts) were excluded from the analysis before identifying differentially expressed genes. Data normalization, dispersion estimates and fitting of a negative binomial model were be carried out with the DESeq2 Bioconductor package.[8] The variance stabilized transformed normalized gene expression of the 500 most variable genes was used to perform a principal component analysis. The Benjamini-Hochberg False Discovery Rate (FDR) procedure was used to re-estimate the adjusted p-values. FDR-corrected p-values (FDR-p) <0.05 were considered significant.

Gene set enrichment analysis (GSEA) was performed to identify enriched biological pathways using the *clusterProfiler* package in R.[9] Genes were ranked according to the Log2 fold-change in expression. Kyoto Encyclopedia of Genes and Genomes[10] gene sets (KEGG) were imported from the Molecular Signatures Database[11] (MSigDB) and a custom tertiary lymphoid structure (TLS) gene set was created comprised of previously described TLS-associated genes.[12]

Example count matrix and code. Code shown in blue

Count Matrix (first four columns and subset of genes shown)

## Gene	Samp1	Samp2	Samp3	Samp4
## TSPAN6	618	476	1588	321
## TNMD	168	89	404	14
## DPM1	828	941	814	897
## SCYL3	529	564	546	440
## C1orf112	155	189	112	144
## FGR	3346	2449	268	5802

DESeq2 Data Matrix for Differential Gene Expression analysis

```
# Creating DESeq dataset (dds)
    library(DESeq2)
    dds <- DESeqDataSetFromMatrix(count_matrix, new.metadata, ~Conditions)

# Count filtration
    idx <- rowSums( counts(dds) >= 5 ) >= 10
    dds <- dds[idx,]

# Setting the baseline comparison and DESeq execution
    dds$Conditions <- relevel(dds$Conditions, "Cont")
    dds <- DESeq(dds)

# Variance Stabilizing Transformation of the count matrix
    rld <- vst(dds, blind = FALSE)
```

Note: For IFA vs IFA+Peptide analysis: DESeq2 performed as above but only including IFA and IFA+Peptide conditions.

PCA of vst transformed counts was performed using *prcomp*

```
pca <- prcomp(t(assay(rld)), center = TRUE)
scores <- as.data.frame(pca$x)
PC1 <- scores$PC1
PC2 <- scores$PC2
PC3 <- scores$PC3
PC4 <- scores$PC4
```

# PC1 vs PC2 and PC3 vs PC4 were then plotted using ggplot2.

GSEA Analysis

# geneList is a list with Log2Fold change and Gene Name for GSEA which was generated by
# DESeq2. This was the comparison of IFA alone and IFA + P.

```
library(clusterProfiler)
library(msigdbr)
```

# Downloading KEGG sets from msigdb

```
m_t2g <- msigdbr(species = "Homo sapiens", category = "C2", subcategory = "CP:KEGG") %>%
dplyr::select(gs_name, entrez_gene)
```

# Sort gene list by Log2FoldChange for GSEA and run GSEA

```
geneList<-sort(geneList, decreasing=TRUE)
em <- GSEA(geneList, TERM2GENE = m_t2g, pvalueCutoff = 0.05, verbose = FALSE)
```

# Plot GSEA results by pathway. This example code shows the generation of the GSEA plot of the most highly enriched set.

```
library(enrichplot)
gseaplot2(em, geneSetID = 1, title = em$Description[1], color = "green")
```

REFERENCES

1. Slingluff CL, Jr., Yamshchikov G, Neese P, et al. Phase I trial of a melanoma vaccine with gp100(280-288) peptide and tetanus helper peptide in adjuvant: immunologic and clinical outcomes. *Clin Cancer Res* 2001;7(10):3012-24. [published Online First: 2001/10/12]

2. Salerno EP, Shea SM, Olson WC, et al. Activation, dysfunction and retention of T cells in vaccine sites after injection of incomplete Freund's adjuvant, with or without peptide. *Cancer Immunol Immunother* 2013;62(7):1149-59. doi: 10.1007/s00262-013-1435-5 [published Online First: 2013/05/10]

3. Melssen M, Petroni G, Grosh W, et al. A multipeptide vaccine plus toll-like receptor (TLR) agonists LPS or polyICLC in combination with incomplete Freund’s adjuvant (IFA) in melanoma patients. 31st Annual Meeting and Associated Programs of the Society for Immunotherapy of Cancer (SITC 2016). National Harbor, MD, 2016:Poster 352.

4. Patel SP, Petroni GR, Gaughan EM, et al. Phase I/II trial of a long peptide vaccine (LPV7) plus toll-like receptor (TLR) agonists for resected stage IIB-IV melanoma. *Journal of Clinical Oncology* 2018;36(15_suppl):e15171-e71. doi: 10.1200/JCO.2018.36.15_suppl.e15171

5. Slingluff CL, Jr., Petroni GR, Olson W, et al. Helper T cell responses and clinical activity of a melanoma vaccine with multiple peptides from MAGE and melanocytic differentiation antigens. *Journal of Clinical Oncology* 2008;26(30):4973-80. doi: 10.1200/JCO.2008.17.3161

6. Slingluff CL, Jr., Petroni GR, Chianese-Bullock KA, et al. Trial to evaluate the immunogenicity and safety of a melanoma helper peptide vaccine plus incomplete Freund's adjuvant, cyclophosphamide, and polyICLC (Mel63). *J Immunother Cancer* 2021;9(1) doi: 10.1136/jitc-2020-000934 [published Online First: 2021/01/23]

7. Pollack KE, Meneveau MO, Melssen MM, et al. Incomplete Freund's adjuvant reduces arginase and enhances Th1 dominance, TLR signaling and CD40 ligand expression in the vaccine site microenvironment. *J Immunother Cancer* 2020;8(1) doi: 10.1136/jitc-2020-000544 [published Online First: 2020/05/01]

8. Love MI, Huber W, Anders S. Moderated estimation of fold change and dispersion for RNA-seq data with DESeq2. *Genome Biol* 2014;15(12):550. doi: 10.1186/s13059-014-0550-8 [published Online First: 2014/12/18]

9. Subramanian A, Tamayo P, Mootha VK, et al. Gene set enrichment analysis: A knowledge-based approach for interpreting genome-wide expression profiles. *Proceedings of the National Academy of Sciences* 2005;102(43):15545-50. doi: 10.1073/pnas.0506580102

10. Kanehisa M, Goto S. KEGG: kyoto encyclopedia of genes and genomes. *Nucleic Acids Res* 2000;28(1):27-30. doi: 10.1093/nar/28.1.27 [published Online First: 1999/12/11]

11. Liberzon A, Subramanian A, Pinchback R, et al. Molecular signatures database (MSigDB) 3.0. *Bioinformatics* 2011;27(12):1739-40. doi: 10.1093/bioinformatics/btr260

12. Melssen MM, Pollack KE, Meneveau MO, et al. Characterization and comparison of innate and adaptive immune responses at vaccine sites in melanoma vaccine clinical trials. *Cancer Immunology, Immunotherapy* 2021 doi: 10.1007/s00262-020-02844-w

13. Behrends S, Vehse K. The β2 Subunit of Soluble Guanylyl Cyclase Contains a Human-Specific Frameshift and Is Expressed in Gastric Carcinoma. *Biochemical and Biophysical Research Communications* 2000;271(1):64-69. doi: https://doi.org/10.1006/bbrc.2000.2596

14. Covino DA, Gauzzi MC, Fantuzzi L. Understanding the regulation of APOBEC3 expression: Current evidence and much to learn. *Journal of Leukocyte Biology* 2018;103(3):433-44. doi: https://doi.org/10.1002/JLB.2MR0717-310R

15. Ho M-F, Lummertz da Rocha E, Zhang C, et al. TCL1A, a novel transcription factor and a co-regulator of NF-κB p65: SNP and estrogen-dependence. *Journal of Pharmacology and Experimental Therapeutics* 2018:jpet.118.247718. doi: 10.1124/jpet.118.247718

16. Mandal S, Curtis L, Pind M, et al. S100A7 (psoriasin) influences immune response genes in human breast cancer. *Experimental Cell Research* 2007;313(14):3016-25. doi: https://doi.org/10.1016/j.yexcr.2007.03.020

17. Bots M, Medema JP. Serpins in T cell immunity. *Journal of Leukocyte Biology* 2008;84(5):1238-47. doi: https://doi.org/10.1189/jlb.0208140

18. Rajagopalan S, Moyle MW, Joosten I, et al. DNA-PKcs controls an endosomal signaling pathway for a proinflammatory response by natural killer cells. *Sci Signal* 2010;3(110):ra14. doi: 10.1126/scisignal.2000467 [published Online First: 2010/02/25]

19. Liu M, Guo S, Stiles JK. The emerging role of CXCL10 in cancer (Review). *Oncol Lett* 2011;2(4):583-89. doi: 10.3892/ol.2011.300 [published Online First: 2012/08/01]

20. Kyoreva M, Li Y, Hoosenally M, et al. CYP1A1 Enzymatic Activity Influences Skin Inflammation Via Regulation of the AHR Pathway. *Journal of Investigative Dermatology* 2020 doi: https://doi.org/10.1016/j.jid.2020.11.024

21. Schotte R, Rissoan MC, Bendriss-Vermare N, et al. The transcription factor Spi-B is expressed in plasmacytoid DC precursors and inhibits T-, B-, and NK-cell development. *Blood* 2003;101(3):1015-23. doi: 10.1182/blood-2002-02-0438 [published Online First: 2002/10/24]

22. Lyu L, Yao J, Wang M, et al. Overexpressed Pseudogene HLA-DPB2 Promotes Tumor Immune Infiltrates by Regulating HLA-DPB1 and Indicates a Better Prognosis in Breast Cancer. *Frontiers in Oncology* 2020;10(1245) doi: 10.3389/fonc.2020.01245

23. Wikenheiser DJ, Stumhofer JS. ICOS Co-Stimulation: Friend or Foe? *Front Immunol* 2016;7(304) doi: 10.3389/fimmu.2016.00304

24. Kubach J, Lutter P, Bopp T, et al. Human CD4+CD25+ regulatory T cells: proteome analysis identifies galectin-10 as a novel marker essential for their anergy and suppressive function. *Blood* 2007;110(5):1550-58. doi: 10.1182/blood-2007-01-069229

25. Sawant KV, Poluri KM, Dutta AK, et al. Chemokine CXCL1 mediated neutrophil recruitment: Role of glycosaminoglycan interactions. *Scientific Reports* 2016;6(1):33123. doi: 10.1038/srep33123

26. Mayer WE, O'HUigin C, Klein J. Resolution of the HLA-DRB6 puzzle: a case of grafting a de novo-generated exon on an existing gene. *Proc Natl Acad Sci U S A* 1993;90(22):10720-4. doi: 10.1073/pnas.90.22.10720 [published Online First: 1993/11/15]

27. Davis AP, Capecchi MR. Axial homeosis and appendicular skeleton defects in mice with a targeted disruption of hoxd-11. *Development* 1994;120(8):2187-98. [published Online First: 1994/08/01]

28. Oganesian A, Poot M, Daum G, et al. Protein tyrosine phosphatase RQ is a phosphatidylinositol phosphatase that can regulate cell survival and proliferation. *Proceedings of the National Academy of Sciences* 2003;100(13):7563-68. doi: 10.1073/pnas.1336511100

29. Zhu Y, Kakinuma N, Wang Y, et al. Kank proteins: a new family of ankyrin-repeat domain-containing proteins. *Biochim Biophys Acta* 2008;1780(2):128-33. doi: 10.1016/j.bbagen.2007.09.017 [published Online First: 2007/11/13]

30. Duda P, Janczara J, McCubrey JA, et al. The Reverse Warburg Effect Is Associated with Fbp2-Dependent Hif1α Regulation in Cancer Cells Stimulated by Fibroblasts. *Cells* 2020;9(1):205.
